# Novel Adult-Onset Systolic Cardiomyopathy Due to MYH7 E848G Mutation in Patient-Derived Induced Pluripotent Stem Cells

**DOI:** 10.1016/j.jacbts.2018.08.008

**Published:** 2018-12-31

**Authors:** Kai-Chun Yang, Astrid Breitbart, Willem J. De Lange, Peter Hofsteen, Akiko Futakuchi-Tsuchida, Joy Xu, Cody Schopf, Maria V. Razumova, Alex Jiao, Robert Boucek, Lil Pabon, Hans Reinecke, Deok-Ho Kim, J. Carter Ralphe, Michael Regnier, Charles E. Murry

**Affiliations:** a Department of Medicine/Cardiology, University of Washington, Seattle, Washington; b Center for Cardiovascular Biology, University of Washington, Seattle, Washington; c Department of Pathology, University of Washington, Seattle, Washington; d Institute for Stem Cell and Regenerative Medicine, University of Washington, Seattle, Washington; e Department of Pediatrics, University of Wisconsin School of Medicine and Public Health, Madison, Wisconsin; f Department of Bioengineering, University of Washington, Seattle, Washington; g Department of Pediatrics, Seattle’s Children’s Hospital and the University of Washington, Seattle, Washington

**Keywords:** disease-modeling, engineered heart tissue, genetic cardiomyopathy, induced pluripotent stem cells, Ad-GFP, green fluorescent protein–encoding adenovirus, cMyBP-C, cardiac myosin-binding protein C, DCM, dilated cardiomyopathy, EHT, engineered heart tissue, FCM, familial cardiomyopathy, HCM, hypertrophic cardiomyopathy, hiPSC-CM, human induced pluripotent stem cell–derived cardiomyocyte, iPSC-CM, induced pluripotent stem cell–derived cardiomyocyte, KO, knockout, MOI, multiplicity of infections, MYH, myosin heavy chain, WT, wild-type

## Abstract

A novel myosin heavy chain 7 mutation (E848G) identified in a familial cardiomyopathy was studied in patient-specific induced pluripotent stem cell–derived cardiomyocytes. The cardiomyopathic human induced pluripotent stem cell–derived cardiomyocytes exhibited reduced contractile function as single cells and engineered heart tissues, and genome-edited isogenic cells confirmed the pathogenic nature of the E848G mutation. Reduced contractility may result from impaired interaction between myosin heavy chain 7 and cardiac myosin binding protein C.

## Highlights

• Many cardiomyopathy families have genetic variants whose significance is unknown. We studied a novel (E848G) mutation in MYH7, a sarcomeric protein.
• Patient-specific induced pluripotent stem cell–derived cardiomyocytes and engineered heart tissues recapitulated the contractile dysfunction.
• Overexpression of the E848G allele in MYH7-null induced pluripotent stem cell–derived cardiomyocytes confirms the causality of the E848G variant.
• The E848G allele disrupts the protein–protein interaction between MYH7 and cardiac myosin binding protein C, presenting a potential mechanism of action.
• Assessing the pathogenicity of new MYH7 variants by overexpressing them in a null background should accelerate their screening for disease causality.

## Visual Abstract

**Figure:**
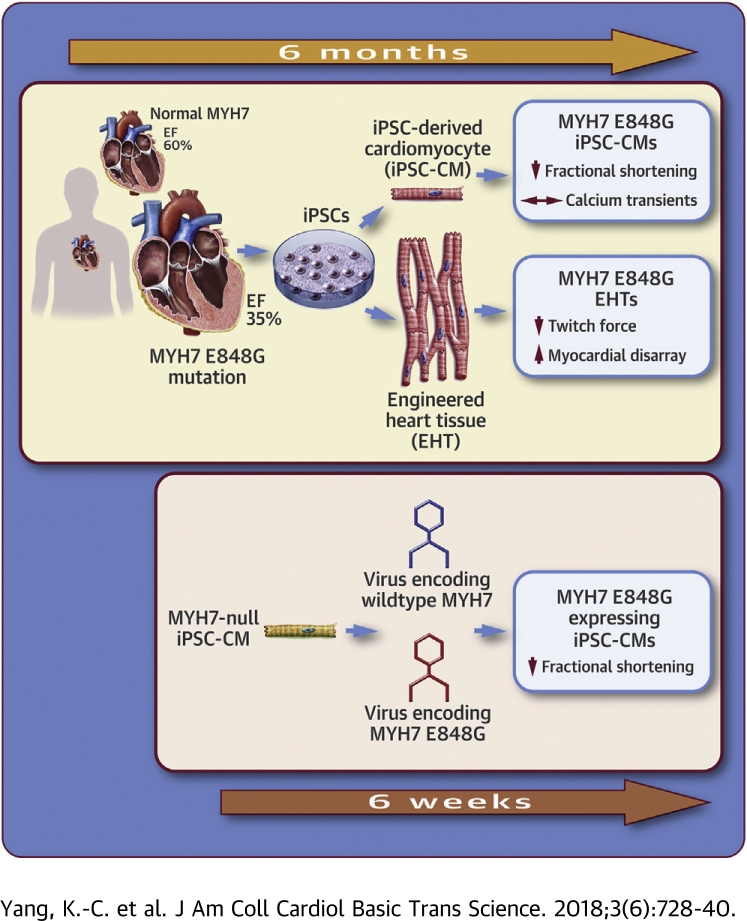

Heart failure affects ∼5 million Americans (1), and two-thirds of the cases are associated with systolic dysfunction (2). Recent advances in genetic testing have identified mutations in >30 genes as causes for systolic dysfunction, with many of these genes encoding proteins within the cardiac sarcomere (3). Despite advances in our understanding of genetic cardiomyopathies, the tools to study these diseases are imperfect. Primary human cardiomyocytes are difficult to obtain and maintain in culture. Traditional mouse models are limited, because the electrical and contractile properties are significantly different to allow for a resting heart rate that is 10-fold higher than in humans. Studies involving β-myosin heavy chain (MYH7) in mice are particularly difficult, as the predominant myosin isoform in mice is α-myosin heavy chain (MYH6).

Human induced pluripotent stem cells (hiPSCs) provide a model to circumvent many of these issues by allowing genetic diseases to be studied in a human context. Recent studies using patient-specific iPSC-derived cardiomyocytes (iPSC-CMs) from individuals with various diseases such as long QT syndrome, Brugada syndrome, hypertrophic cardiomyopathy (HCM), and dilated cardiomyopathy (DCM) have recapitulated many features of the human disease and have advanced our knowledge of the pathophysiology in human cells 4, 5.

Although iPSC technology is promising, it can be difficult to model genetic cardiac diseases that have their onset in adulthood due to the immaturity of iPSC-CMs. In addition, proving that a novel variant causes the disease can be difficult using statistical genetics when only small kindreds are available. In the current study, we encountered both of these challenges when studying an African-American familial cardiomyopathy (FCM) with adult-onset systolic dysfunction associated with a rare MYH7 E848G variant (rs727504311). This variant is not described in large population databases such as the Exome Aggregation Consortium or the Genome Aggregation Database but has recently been described by the ClinVar database as “likely pathogenic” (6). Although contractile deficits were not apparent in early iPSC-CMs carrying the E848G allele, when cells were matured by prolonged culture time or via tissue engineering, their contractile deficits were readily apparent. Using genetic editing and viral transgenesis, we established that MYH7 with the E848G mutation reduces contractile function in a dominant-negative manner, and we provided evidence that a reduced ability to bind cardiac myosin-binding protein C (cMyBP-C) may contribute to the contractile dysfunction.

## Methods

Details of the materials and methods are available in the Supplemental Material. Briefly, all patient recruitments followed the protocol approved by the institutional review board. Dermal fibroblasts from FCM and normal healthy patients were episomally reprogrammed to iPSCs and differentiated into cardiomyocytes with our well-established monolayer differentiation protocol (7). Patient-specific iPSC-CMs were replated as single cells or in a three-dimensional co-culture to form engineered heart tissues (EHTs) to assess contractile function. The Clustered Regularly Interspaced Short Palindromic Repeats/CRISPR-Associated Protein 9 system was used to knock out MYH7 (MYH7 KO) to create an isogenic iPSC line. Yeast two-hybrid analysis was used to test the protein–protein interaction between MYH7 S2 and cMyBP-C C1C2 domains.

## Results

### Early-onset systolic impairment in an FCM family associated with MYH7 E848G mutation

**Figure 1:**
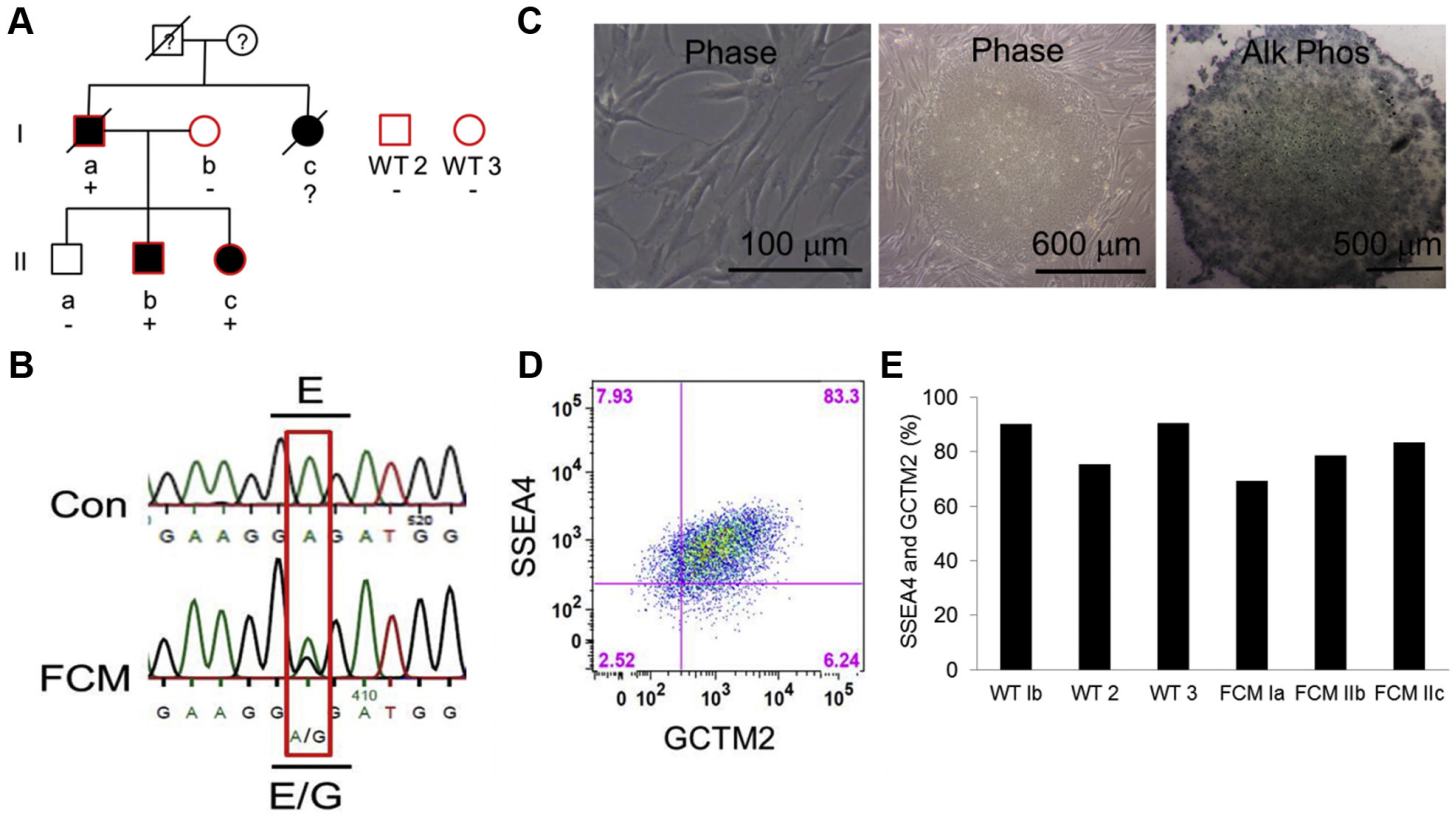
Derivation and Characterization of Patient-Specific FCM iPSC-CMs **(A)** Pedigree of the familial cardiomyopathy (FCM) with the myosin heavy chain 7 (MYH7) E848G missense mutation in this study. **Solid symbols** indicate echocardiographic evidence of systolic impairment; “**+**” and “**-**” represent the presence and absence of the MYH7 E848G mutation, respectively. The **red border** indicates the inclusion of the individual’s induced pluripotent stem cells (iPSCs) in this study. **(B)** Deoxyribonucleic acid sequencing confirms MYH7 E848G heterozygous mutation in the FCM patients and the absence of the mutation in the control (Con) subjects. **(C)** Representative images of fibroblasts from a skin biopsy sample **(left)**, the iPSC colony obtained after episomally reprogramming dermal fibroblasts **(middle)**, and alkaline phosphatase (Alk Phos) staining of the patient-specific iPSC colony **(right)**. **(D)** Representative flow cytometry of 2 pluripotency markers, SSEA4 and GCTM2, in an iPSC line with **(E)** quantification of double-positive population in all 6 patient-specific iPSC lines. CM = cardiomyocytes; Phase = phase microscopy; WT = wild type.

Individual FCM Ia presented at 44 years of age after being resuscitated from sudden cardiac death. He was found to have a left ventricular ejection fraction of 35% with normal wall thickness and a nondilated ventricle on echocardiogram (Figure 1A, Supplemental Tables 1 and 2). His sister (Ic) presented with congestive heart failure symptoms at 63 years of age and a left ventricular ejection fraction of 31% with normal wall thickness and a nondilated ventricle. She subsequently died of heart failure but was never genotyped. Both patients (Ia and Ic) had normal epicardial coronaries on angiography at time of presentation. Due to the lack of ventricular hypertrophy or chamber dilation, they did not meet the criteria for HCM or DCM, and the etiology of their cardiomyopathy was otherwise unclear. Direct exon sequencing of 9 genes (MYH7, MYBPC3, TNNT2, TNNI3, ACTC, MYL2, MYL3, LAMP2, and PRKAG2) identified a novel heterozygous missense mutation that caused a glutamic acid-to-glycine mutation at amino acid position 848 (E848G) on MYH7 in the proband (Patient Ia). The offspring were subsequently specifically tested for the E848G allele only. This mutation occurs at a residue conserved from mouse to humans (Supplemental Table 3).

Unlike the older generation, who had overt systolic dysfunction with reduced ejection fraction, screening echocardiograms for 3 children in the younger generation (IIa, IIb, and IIc) identified markedly decreased systolic wall thickening of the interventricular septum with preserved ejection fraction in the 2 individuals with the MYH7 E848G allele, and normal contractile function in the wild-type (WT) individual (Figures 1A and 1B, Supplemental Table 1). Systolic wall thickening is a regional measure of systolic function 8, 9 and has been shown to be sensitive for the detection of regional contractile abnormalities despite globally preserved left ventricular ejection fraction (8).

### Derivation and characterization of patient-specific iPSCs

Human iPSCs were generated from 3 affected individuals (FCM Ia, IIb, and IIc) and 3 healthy individuals (WT Ib and unrelated subjects 2 and 3) by reprogramming their skin fibroblasts. Pluripotent colonies were chosen based on colony morphology, staining for alkaline phosphatase, and co-expression of the pluripotency markers SSEA4 and GCTM2 (Figures 1C to 1E).

### Generation of patient-specific iPSC-CMs with aligned myofibrils

All patient-specific iPSC lines were directly differentiated into cardiomyocytes that were >60% cardiac troponin T–positive according to flow cytometry. Myofibril alignment of neonatal rat cardiomyocytes has been shown to be significantly improved by plating cells onto nanopatterned surfaces with parallel grooves/ridges of 800 nm width and spacing (10). We reasoned that aligning the myofibrils of our iPSC-CMs would minimize noise in contractile measurements due to myofibril disarray. We plated patient-derived iPSC-CMs onto nanopatterned surfaces and found dramatically improved myofibril alignment 5 days later compared with nonpatterned surfaces (Supplemental Figure 1A). After a period of 30 days, we observed that the myofibrils in all 6 lines of iPSC-CMs (3 WT and 3 FCM) aligned within 15 degrees of the orientation of the nanopatterned substratum (Supplemental Figure 1B, Supplemental Table 4). There was no difference in cell size between groups (Supplemental Figure 2). Overall, a nanopatterned surface structurally matured the iPSC-CMs and transformed their contractile cytoskeletons into a more adult cardiomyocyte-like phenotype.

### FCM iPSC-CMs develop impaired fractional shortening over time

IonOptix optical video microscopy (IonOptix LLC, Westwood, Massachusetts) was used to assess contractile properties of individual hiPSC-CMs. Longitudinal cell edges or cytoplasmic granules of isolated hiPSC-CMs on nanopatterned surfaces were tracked with 1.5 Hz pacing (Figure 2, Supplemental Videos 1 and 2). At 30 days’ post-differentiation, the FCM-CMs had no differences in fractional shortening compared with WT-CMs (Figure 2A). The day 30 FCM-CMs had a minimally (∼15%; p < 0.05) increased maximal rate of contraction (Supplemental Figure 3A) and showed no differences in maximal rate of relaxation, time to (25%, 50%, 75%, and 100%) peak contraction, or time to (25%, 50%, and 75%) baseline (Supplemental Figures 3B and 3C). Thus, there was no overt phenotype seen in the E848G cardiomyocytes at 30 days’ post-differentiation.Figure 2Characterization of Contractile Function and Calcium Transients in hiPSC-CMs**(A)** No difference in maximum averaged normalized shortening in day 30 WT-CMs and FCM-CMs. **(B, C)** Impaired maximum fractional shortening is evident in day 50 FCM-CMs compared with age-matched WT-CMs. (N = 3 WT lines and N = 3 FCM lines, n > 10 cells for each day 30 line; Kruskal-Wallis test, p > 0.05) (N = 3 WT lines and N = 3 FCM lines, n > 21 for each day 50 line; Kruskal-Wallis test, p < 0.05; *p < 0.05 with 2-tailed Mann-Whitney *U* test). **(D)** Normalized averaged calcium transients in day 50 human induced pluripotent stem cell–derived cardiomyocyte (hiPSC-CMs) are not significantly different. All cells paced at 1.5 Hz. Error bars indicate SEM. See Supplemental Videos 1 and 2. Abbreviations as in Figures 1 and 2.
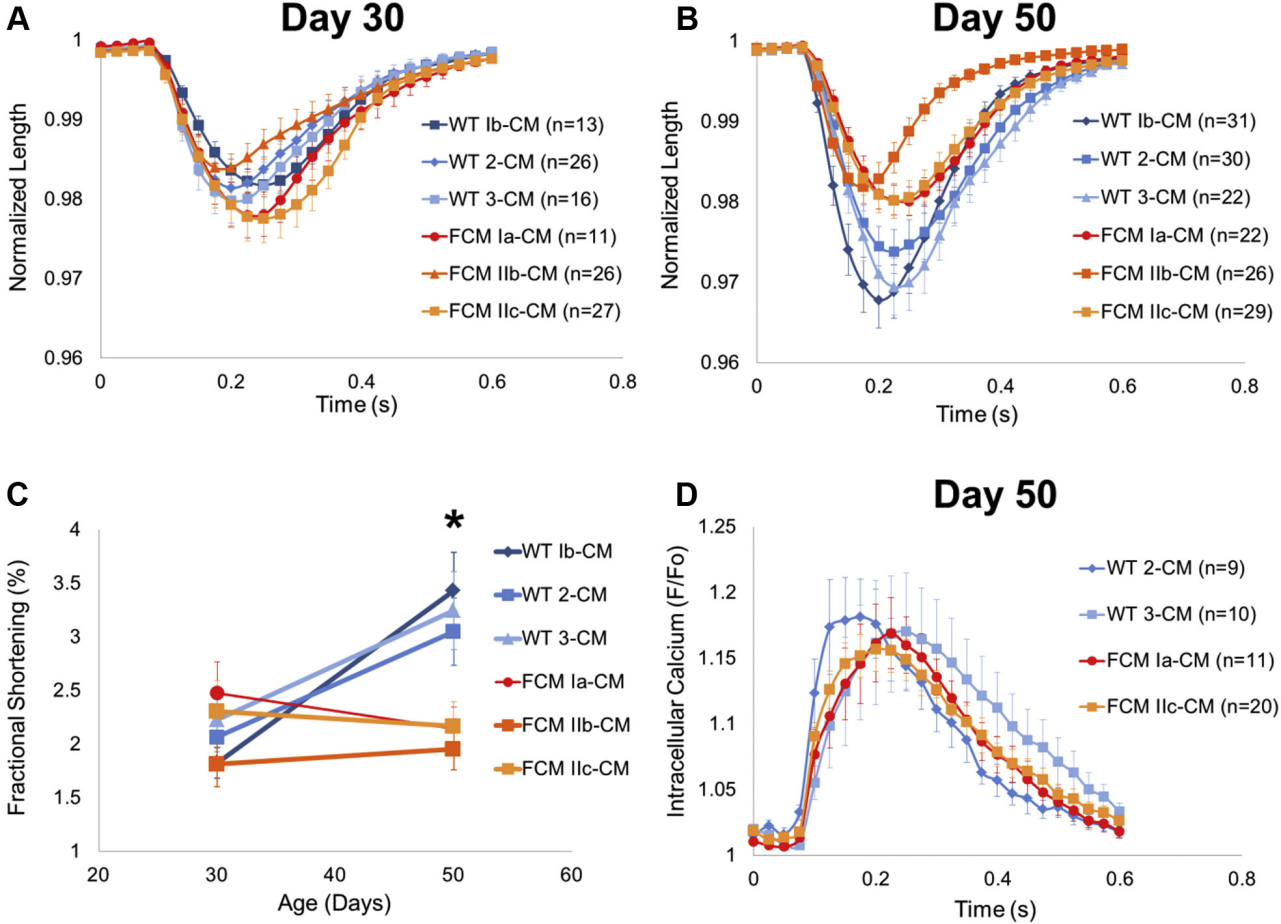

**Figure:**
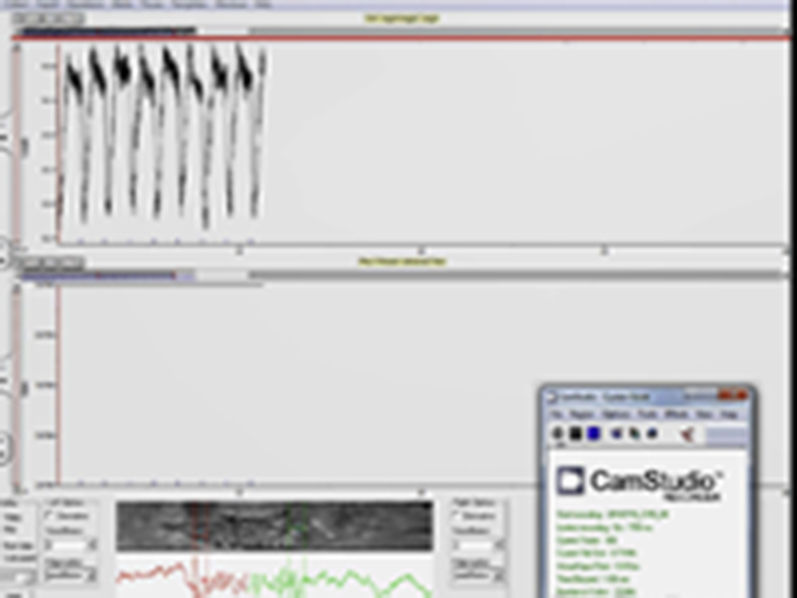
Supplemental Video 1 Representative IonOptix video recording of a hiPSC-CM plated on a nanopatterned surface and contracting at 1.5 Hz.

**Figure:**
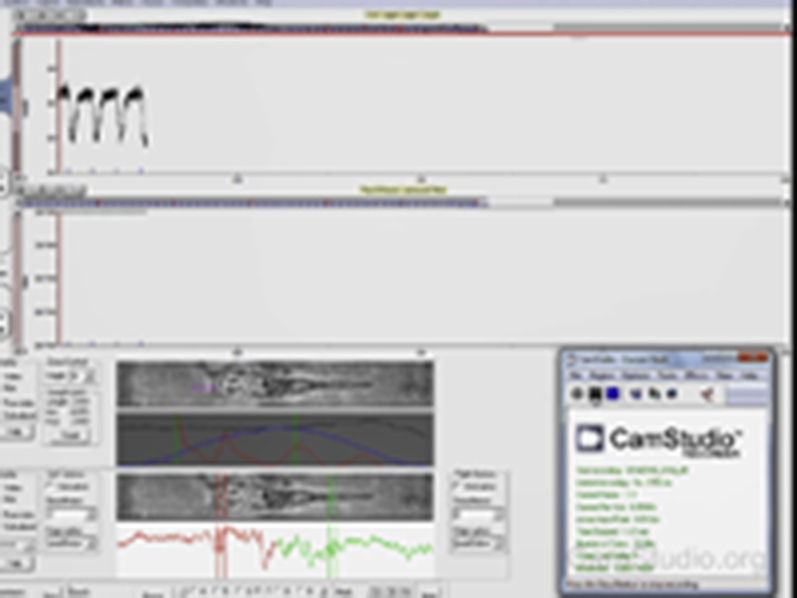
Supplemental Video 2 Another representative IonOptix video recording of a hiPSC-CM plated on a nanopatterned surface and contracting at 1.5 Hz.

Given that the most striking phenotype in this family was overt systolic dysfunction during mid-life, we hypothesized that maturing the FCM iPSC-CMs on the nanopatterns over time would reveal the contractile dysfunction seen in these patients. In support of this hypothesis, when the cells were aged out to 50 days, fractional shortening of WT lines improved from 2.04 ± 0.20% to 3.24 ± 0.19%, whereas the fractional shortening of age-matched FCM-CMs remained constant, from 2.20 ± 0.34% to 2.09 ± 0.12% (N = 3 WT lines and N = 3 FCM lines, n > 11 cells for each line; Kruskal-Wallis test, p < 0.05; post hoc Mann-Whitney *U* test, p < 0.05 between day 50 WT-CMs and FCM-CMs) (Figures 2B and 2C). No difference was seen in maximal rate of contraction, maximal rate of relaxation, time to (25%, 50%, 75%, and 100%) peak contraction, or time to (25%, 50%, and 75%) baseline in the day 50 FCM-CMs (Supplemental Figures 3D to 3F). Thus, WT hiPSC-CMs increased their systolic shortening with maturation in vitro, whereas the FCM iPSC-CMs failed to do so.

**Figure 3:**
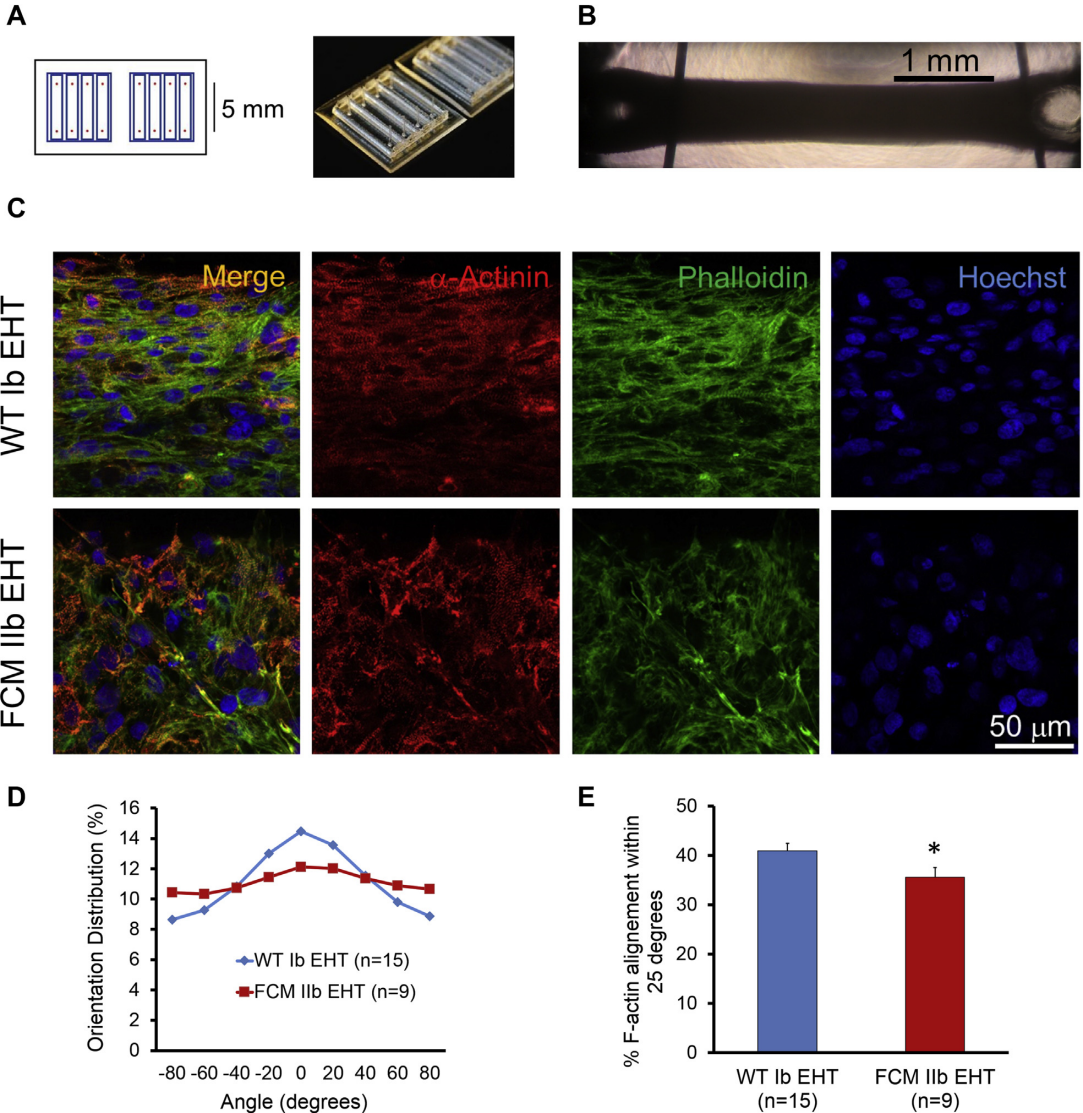
Generation and Characterization of Patient-Specific EHTs **(A)** Schematic **(left)** and photograph **(right)** of polydimethylsiloxane molds used to create engineered heart tissue (EHTs). **(B)** Bright-field image of EHTs in culture. Vertical bars are pins used to prevent EHTs from migrating out of the mold. **(C)** EHT immunofluorescence with α-actinin, f-actin (phalloidin), and nuclear staining. **(D, E)** Quantitative analysis of cytoskeletal alignment of EHTs conditioned with static stress. Vector of stress conditioning is parallel to EHT edge (horizontal). *p < 0.05 (Student’s *t*-test). Error bars indicate SEM.

### No difference in day 50 FCM iPSC-CM calcium transients

To assess whether the differences in fractional shortening were due to changes in calcium handling, calcium transients were measured by using the ratiometric dye fura-2 with the IonOptix system. Despite the differences in fractional shortening seen in day 50 FCM-CMs, no difference was observed in maximal intracellular calcium, maximal rate of calcium decay, or time to baseline calcium between 2 WT-CM lines and 2 FCM-CM lines (Figure 2D, Supplemental Figure 4). Thus, differences in contractile function are likely to reside in the myofibrils rather than in the elements of excitation-contraction coupling.

### Impaired force generation in patient-specific EHTs

We next tested whether the impaired fractional shortening on the cellular level translated into contractile dysfunction on the tissue level. We made EHT in a three-dimensional co-culture by mixing either age-matched WT Ib or FCM IIb hiPSC-CMs with human marrow stromal cells into custom polydimethylsiloxane molds with troughs measuring 8 × 2 mm (Figure 3A) as previously described (11). Posts were designed to be 5 mm apart to form loops on either end of the EHT (Figure 3B). Constructs were conditioned with static external stress for 2.5 to 3 weeks to increase cellular alignment and hypertrophy. Interestingly, EHTs with WT hiPSC-CMs exhibited significantly greater cardiomyocyte alignment than did those from FCM hiPSC-CMs (p < 0.05) (Figures 3C to 3E).

**Figure 4:**
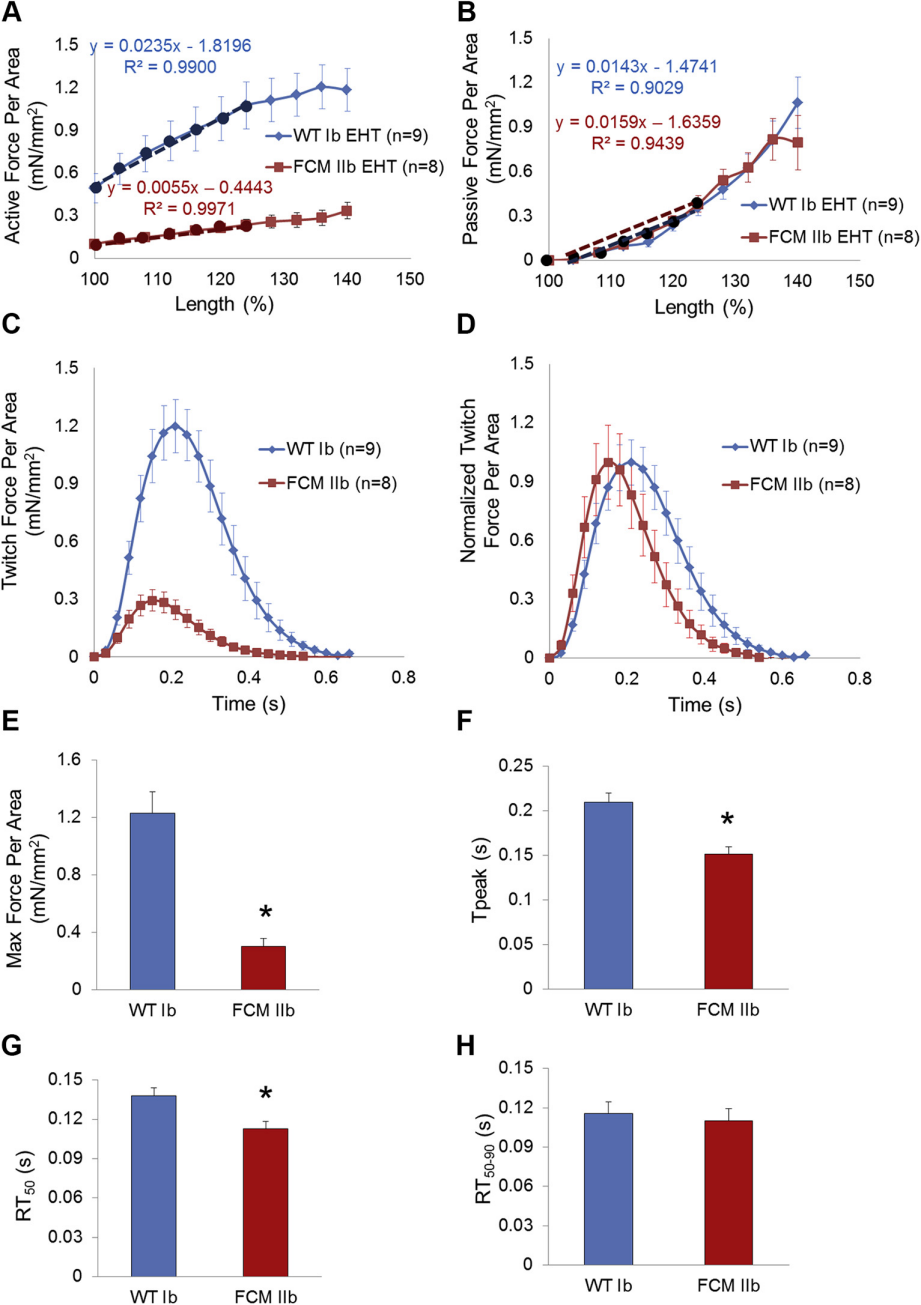
Force-Length Curve and Maximal Twitch Force Production in Patient-Specific EHTs WT Ib-CM and FCM IIb-CM were used to generate patient-specific EHTs. After attaching EHTs to the force transducer, **(A)** active force per area twitch height was graphed against EHT length (Starling curve). The active force per area increased in a linear fashion over increasing preparation lengths. The slope of the first 25% length change **(dotted line)** was lower in the FCM EHT compared with the WT EHT (p < 0.0001), indicating impaired contractility in the FCM EHT. **(B)** Passive force per area was graphed against EHT length. The slope of the first 25% length change (**dotted line**, Young’s modulus) was unchanged between the 2 groups (p = 0.57). **(C)** Absolute and **(D)** normalized twitch force per area traces of WT Ib EHT and FCM IIb EHT measured at 140% length. **(E)** Maximum (Max) force per area produced. **(F)** Time to peak contraction (T_peak_). **(G)** T_peak_ to 50% relaxation (RT_50_). **(H)** Time from 50% to 90% relaxation (RT_50–90_). All EHTs were paced at 1.5 Hz, and all force values were normalized to cross-sectional area. *p < 0.05 (Student’s *t*-test). Error bars indicate SEM. Abbreviations as in Figures 1 and 3.

To study isometric contractile function of the patient-specific EHTs, the loops in the EHTs were mounted between a force transducer and a rod whose position was precisely controlled by a length controller. Force was continuously measured while the length controller stretched the EHT to generate passive tension on the EHT. Average active force transients per cross-sectional area (twitch force per area) of 9 WT Ib EHTs and 8 FCM IIb EHTs were plotted against length (Figure 4A, Supplemental Figure 5) to generate Starling curves. The Starling curve slope, a commonly used measure of myocardial contractility, for WT Ib EHTs was 0.0235 mN/mm^2^/% length, whereas the slope for FCM IIb EHTs was 0.0055 mN/mm^2^/% length (analysis of covariance, p < 0.0001). Thus, the contractility of the FCM IIb EHTs was 4-fold lower than that of WT Ib EHTs. The slope of the diastolic tension versus length curve defines passive stiffness (i.e., Young’s modulus). The passive stiffness was comparable between WT Ib and FCM IIb EHTs (0.0143 mN/mm^2^/% vs. 0.0159 mN/mm^2^/% length, respectively; analysis of covariance, p = 0.57) (Figure 4B).

Twitch kinetics and amplitude comparisons are shown in Figures 4C and 4D. Strikingly, the maximal active twitch force per area of the FCM IIb EHT was reduced by >75% compared with WT Ib EHT (p < 0.001) (Figure 4E). When normalized to total force per twitch, both the time from electrical stimulation to maximal force production and to 50% relaxation were shorter in the FCM IIb EHTs compared with the WT Ib EHTs (both, p < 0.05) (Figures 4F and 4G). There was no difference in time from 50% relaxation to time of 90% relaxation (Figure 4H). Together, these studies indicate a significant reduction in systolic function in the E848G-carrying cardiomyocytes, with minimal impact on diastolic function.

### Isogenic studies indicate a dominant-negative role for MYH7 E848G

Initial attempts at using adenoviral transduction to overexpress either WT or E848G MYH7 in healthy hiPSC-CMs revealed no difference in contractile function (Supplemental Figure 6). We suspect these experiments were unsuccessful due to competition from the endogenous WT MYH7. To circumvent this problem, a homozygous knockout of MYH7 was created in a WT hiPSC line using the Clustered Regularly Interspaced Short Palindromic Repeats/CRISPR-Associated Protein 9 system to induce double strand breaks immediately after the start codon in exon 3 of MYH7. Nonhomologous end joining randomly created a 16 nucleotide deletion at this locus, which resulted in a new premature stop codon (Figures 5A and 5B). Karotypically normal homozygous MYH7 knockout (MYH7 KO) iPSCs (Supplemental Figure 7) were differentiated to MYH7 KO iPSC-CMs (MYH7 KO-CMs) that lack MYH7 protein but still form intact sarcomeres (Figures 5C and 5D) and beat spontaneously due to the presence of MYH6 (Figure 5, Supplemental Video 3).Figure 5MYH7 E848G Induces Systolic Dysfunction in Isogenic Line**(A)** Schematic diagram of Clustered Regularly Interspaced Short Palindromic Repeats/CRISPR-Associated Protein 9 system–mediated homozygous MYH7 knockout (KO) in a normal hiPSC line. **(B)** Sequence and chromatogram (homozygote) of the start region of MYH7 KO iPSC. **(C)** Representative immunofluorescent microscopic images reveal formation of sarcomeres despite the absence of MYH7 in MYH7 KO-CMs. **(D)** Immunoblot confirms MYH7 KO in MYH7 KO-CMs and approximately equal amounts of MYH7 protein 3 days after transduction with either 15 multiplicity of infections (MOIs) of Ad-MYH7 or 25 MOI of Ad-E848G. **(E)** Immunofluorescent microscopic images show incorporation of c-term flag-tagged MYH7 and MYH7 E848G into sarcomeres of MYH7 KO-CMs. **(F, G)** MYH7 KO-CMs treated with 25 MOI Ad-E848G demonstrate impaired fractional shortening compared with MYH7 KO-CMs treated with 50 MOI of green fluorescent protein–encoding adenovirus (Ad-GFP) or 15 MOI of Ad-MYH7 (Kruskal-Wallis test, p < 0.05; post hoc 2-tailed Mann-Whitney *U* test, p < 0.05 between Ad-GFP and Ad-E848G; post hoc 2-tailed Mann-Whitney *U* test, p < 0.05 between Ad-MYH7 and Ad-E848G). See Supplemental Video 3. GAPDH = glyceraldehyde-3-phosphate dehydrogenase; other abbreviations as in Figures 1 and 2.
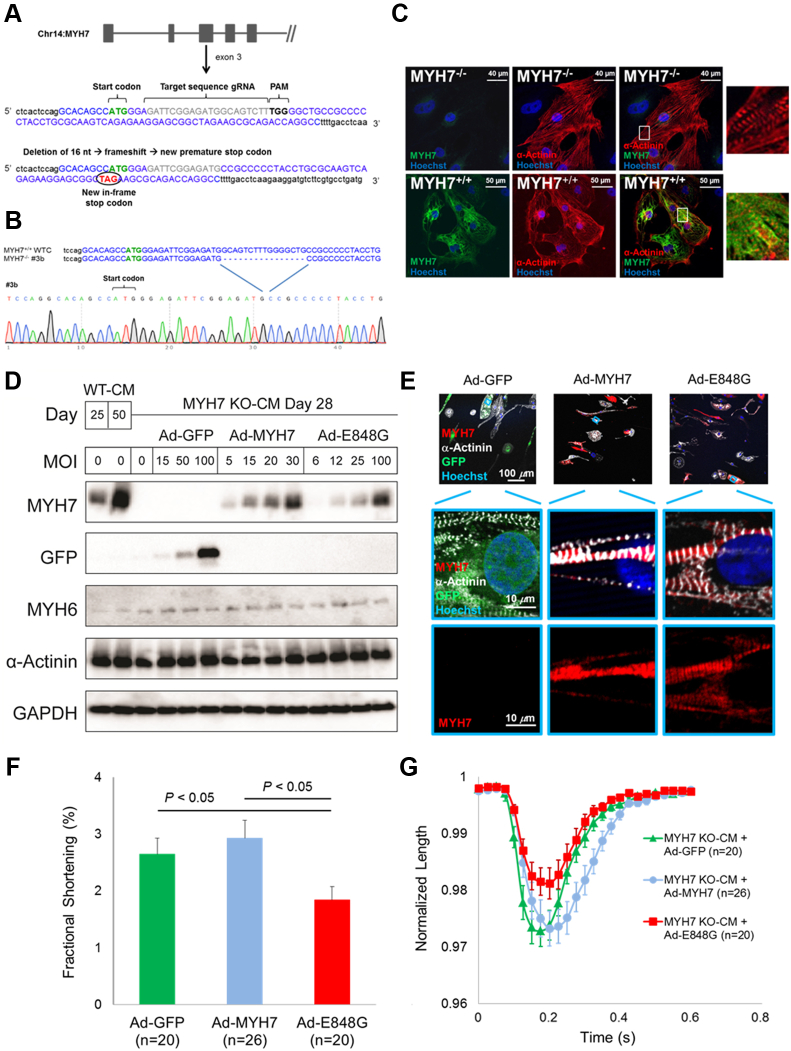

**Figure:**
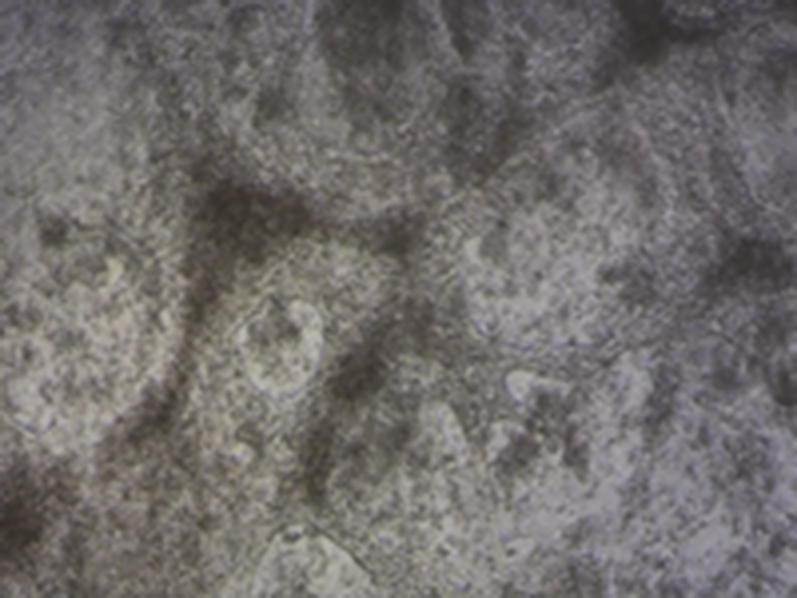
Supplemental Video 3 Video of MYH7 KO-CMs contracting spontaneously.

Because contractile function is dependent on total myosin protein level in addition to genotype, we transduced MYH7 KO-CMs with a range of Ad-MYH7 and Ad-E848G multiplicity of infections (MOIs) and carefully selected viral doses that yielded normal MYH7 protein levels 3 days later (Figure 5D). Control cells received a green fluorescent protein–encoding adenovirus (Ad-GFP). We confirmed successful incorporation of the FLAG-tagged MYH7 and MYH7 E848G proteins into MYH7-null sarcomeres (Figure 5E). Two weeks after viral treatment, MYH7 KO-CMs transduced with Ad-E848G exhibited significantly less fractional shortening (1.84 ± 0.23%) compared with cells treated with Ad-GFP (2.65 ± 0.28%) or Ad-MYH7 (2.93 ± 0.31%) (Kruskal-Wallis test, p < 0.05; post hoc 2-tailed Mann-Whitney *U* test, p < 0.05 between Ad-GFP and Ad-E848G; post hoc 2-tailed Mann-Whitney *U* test, p < 0.05 between Ad-MYH7 and Ad-E848G) (Figures 5F and 5G). This outcome confirms the pathogenic role of MYH7 E848G in mediating this familial systolic cardiomyopathy and suggests that it is acting as a dominant-negative poison peptide.

### MYH7 E848G mutation disrupts MYH7 S2 and cMyBP-C C1C2 interaction

A common HCM mutation associated with systolic dysfunction, cMyBP-C E258K, disrupts a key hydrogen bond between the C1C2 domain of cMyBP-C and the S2 domain of MYH7 at amino acid T857 12, 13, 14. Yeast two-hybrid analysis confirmed that the E258K mutation in cMyBP-C eliminated the protein–protein interaction between the 2 proteins, suggesting that the loss of S2-C1C2 interaction leads to systolic dysfunction (12). Because the MYH7 E848G mutation in our patients is predicted also to disrupt a hydrogen bond between MYH7 E848 (9 amino acids away from T857) and cMyBP-C K195 at the same molecular interface (13), we hypothesized that the contractile impairment seen in FCM iPSC-CMs and FCM EHTs is due to the disruption of the MYH7 S2 and cMyBP-C C1C2 protein–protein interaction. We tested this hypothesis in a yeast two-hybrid system. The S2 domains of WT and mutant human MYH7 were fused to the GAL4-activation domain (S2^WT^/S2^E848G^-AD, respectively). Similarly, the C1C2 domains of WT and mutant human cMyBP-C were fused to the GAL4–deoxyribonucleic acid binding domain (C1C2^WT^/C1C2^E258K^-BD, respectively). Because myosin S2 is known to form coiled-coil dimers 12, 15, the S2^WT^-BD fusion peptide was also constructed and used to assess possible effects of the E848G mutation on S2 homodimerization. Coexpression of BD and AD fusion peptides was performed with yeast mating, and protein–protein interaction was assessed by the ability of the yeast to grow on minimal selection media.

**Figure 6:**
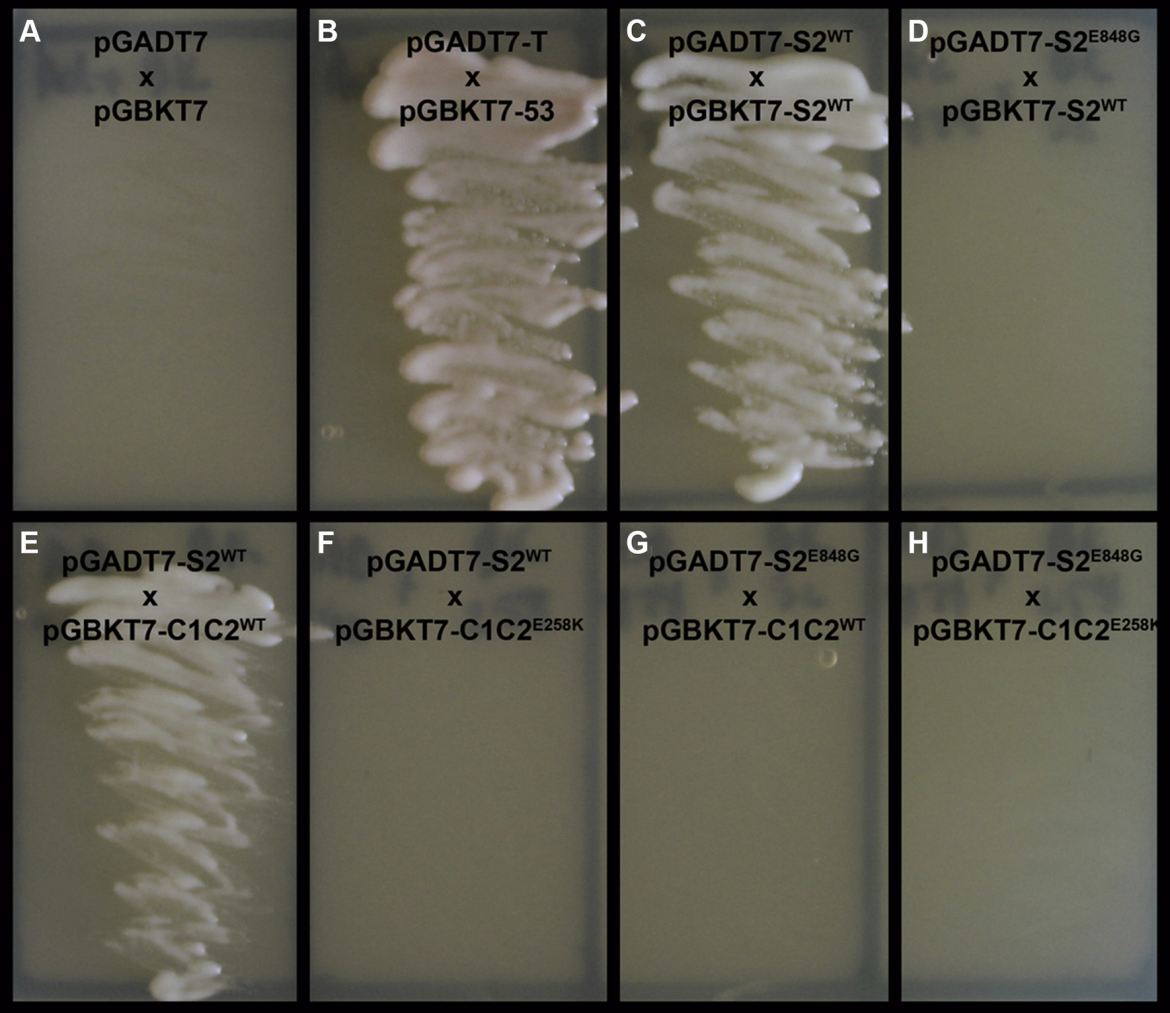
Effect of the S2 E848G Mutation on the Interaction Between Myosin S2 and N-Terminal cMyBP-C Yeast two-hybrid interaction tests between the S2 domain of MYH7 and the C1C2 domain of cardiac myosin-binding protein C (cMyBP-C). **(A)** Negative control. **(B)** Positive control. **(C)** Positive S2^WT^-S2^WT^ interaction. **(D)** No S2^E848G^-S2^WT^ interaction. **(E)** Positive S2^WT^-C1C2^WT^ interaction. **(F)** No S2^WT^-C1C2^E258K^ interaction. **(G)** No S2^E848G^-C1C2^WT^ interaction. **(H)** No S2^E848G^-C1C2^E258K^ interaction. **A–H,** Yeast grown on selection media.

As expected for a negative control, no growth was seen with unfused BD and AD peptides (Figure 6A), whereas the positive control (Figure 6B) and S2^WT^-AD and S2^WT^-BD resulted in robust growth, confirming S2 homodimerization (Figure 6C). Interestingly, the S2^E848G^-AD and S2^WT^-BD condition did not have any growth, indicating that the E848G mutation weakens the dimerization of myosin S2 (Figure 6D). S2^WT^-AD and C1C2^WT^-BD resulted in growth, confirming the interactions between MYH7 and cMyBP-C (Figure 6E). The S2^WT^-AD and C1C2^E258K^-BD combination did not have any growth, confirming that this mutation in cMyBP-C prevents its binding to the WT S2 domain (Figure 6F) (12). The key finding of this yeast-two hybrid experiment, however, was the lack of growth with the S2^E848G^-AD and C1C2^WT^-BD fusion proteins (Figure 6G). As expected, the double-mutation experiment involving S2^E848G^-AD and C1C2^E258K^-BD did not result in any growth (Figure 6H). Taken together, these experiments support the hypothesis that the E848G mutation results in disruption of the myosin S2 and cMyBP-C C1C2 protein–protein interaction.

## Discussion

Genetic causes of familial cardiomyopathies such as HCM or DCM have been identified for several decades (16); however, the mechanisms by which mutations in the sarcomeres lead to contractile dysfunction are still unknown. The present paper describes a family with a predominantly systolic cardiomyopathy due to an MYH7 E848G mutation that is characterized by subtle early contractile abnormalities which later lead to overt systolic dysfunction and arrhythmias. Using patient-specific iPSC-CMs as a source to study human disease in a human context, we have matured hiPSC-CMs with anisotropically aligned nanopatterned substrates and 3-dimensional tissue engineering to recapitulate the systolic dysfunction seen in this family. Although fruitful, previous contractile studies in hiPSC-CMs 17, 18, 19, 20 were performed on cells without aligned myofibrils. By conducting contractile measurements on aligned myofibrils, we believe the current findings are more physiologically relevant.

Although WT and mutant hiPSC-CMs replated on nanopatterned surfaces had very similar myofibril alignment, it was interesting that there was significantly less alignment in FCM EHTs compared with WT EHTs. This lack of alignment correlates with the myofiber disarray noted in the pathology report of patient Ic, who had the FCM phenotype but whose genotype is unknown. The pathogenesis of myofiber disarray is unknown, but it is interesting to speculate that it could relate to the impaired homodimerization of the S2 domain of MYH7 we observed by using the yeast two-hybrid analysis. Another possibility is that the alignment in engineered tissues may depend on the overall stress that is applied to the construct. Because the WT Ib EHTs generate more active force, this action may be providing a stronger alignment stimulus parallel to the long-axis of EHT. It is notable that hiPSC-CMs seeded on the nanopatterned surfaces exhibited no genotype-dependent differences in myofibril alignment, which suggests that the 2-dimensional system generates stronger external alignment stimuli than in the 3-dimensional tissue engineering.

A contractile impairment was observed in day 50 FCM-CMs that was not present in the day 30 FCM-CMs. We suspect this finding is in part likely due to the increased expression of MYH7 that occurs in older day 50 hiPSC-CMs compared with the younger day 30 FCM-CMs (review elsewhere [21]) (Figure 5D). Essentially, the MYH7 E848G allele acts as a dose-dependent poison peptide whose effects become more apparent at high expression levels. Although there were relatively modest contractile differences at the single-cell level in 2 dimensions, there were striking differences between genotypes in EHTs. This difference may result in part from the presence of many cells operating in series in the tissue. The serial architecture serves as a multiplier of force generation and may make contractile differences easier to detect. It is important to recognize, however, that the 75% force reduction in FCM EHTs likely would not be compatible with life if it held true in vivo, so the EHT model is not fully recapitulating cardiac development. Other compensatory neurohormonal mechanisms or mechanical stimuli may preserve systolic function that are absent from our in vitro tissue engineering system.

To confirm the pathogenic role of MYH7 E848G in causing this systolic cardiomyopathy, we strategically chose to knock out MYH7 in a WT iPSC line and then rescue it with MYH7-expressing adenoviruses. Although adenoviral cytotoxicity was present at high MOI, we found that at low MOI levels, the cytotoxicity was minimal, and the salient clinical phenotype was recapitulated. This method not only confirms the pathogenic role of MYH7 E848G in mediating this systolic dysfunction, but it also provides a novel isogenic platform that can be adapted for the future study of other MYH7 variants by simply overexpressing other MYH7 mutations. This approach should save considerable time in screening other MYH7 variants, of which there are many (22), compared with performing homology-directed repair to knock in each variant.

The yeast two-hybrid experiments show that the MYH7 E848G mutation disrupts interaction of myosin’s S2 domain with the cMyBP-C C1C2 domain. This hydrogen bond is 1 of the 9 or 10 hydrogen/ionic bonds that are predicted to mediate the S2:C1C2 interaction (13). Loss of another one of these hydrogen bonds with the cMyBP-C E258K mutation has been shown to disrupt S2:C1C2 interaction in a yeast two-hybrid model and lead to contractile impairment (12). Despite being one of the most common HCM mutations, cMyBP-C E258K carriers have a 33% chance of developing systolic dysfunction, which is 3 times the rate of that in patients with HCM in general (14). Given that both MYH7 E848G and cMyBP-C E258K mutations result in decreased force production, this finding suggests that the MYH7 S2 and cMyBP-C C1C2 protein–protein interaction is necessary for the preservation of systolic function; further studies are needed to confirm this hypothesis, however.

### Study limitations

The relatively slow pace of generating hiPSCs, creating isogenic lines, and phenotyping their cardiomyocytes meant that we could only study 3 different FCM and 3 different normal patients. This method is similar to other iPSC disease modeling reports in the field 5, 23, 24, 25. Although our genotypes clustered together clearly with little overlap, it would be advantageous to increase the throughput for analysis. The MYH7 knockout platform we developed should allow for easier adaptation to study other MYH7 mutations by simply creating new MYH7-expressing viruses. Another limitation is the use of hiPSC-CM on a two-dimensional surface. Because the cells are adherent, it is not possible for them to fully shorten, nor can their loading conditions be readily controlled (26). We improved upon the standard two-dimensional analysis by plating hiPSC-CMs on an anisotropically nanopatterned substrate. This method aligned their myofibrils and increased their force vector, which should reduce experimental noise. Nevertheless, this limitation led us to use EHTs, in which true shortening occurs and mechanical loading can be readily controlled. These experiments revealed remarkably weaker twitch forces in FCM EHTs that phenocopied the findings of the cMyBP-C E258K EHT study that used mouse cardiomyocytes (12). The convergence of these 2 mutations on the disruption of MYH7–cMyBP-C binding suggests that this interaction is crucial for contractile function. Finally, although our data strongly suggest that the MYH7 E848G allele is pathogenic, we cannot rule out the possibility that other clinical factors such as hypertension may act as disease modifiers. Although Individual FCM Ia had hypertension (a known risk factor for systolic dysfunction), Individual FCM Ic did not have a history of hypertension and still developed overt systolic dysfunction, albeit in the sixth instead of the fifth decade of life; this development suggests that hypertension may exacerbate the disease but is unlikely the sole reason for the overt systolic dysfunction.

Overall, we report a unique cardiomyopathy due to MYH7 E848G mutation that clinically does not clearly fit a diagnosis of HCM or DCM. Recent research suggests that myosin variants that destabilize the myosin interacting heads motif and prevent the super-relaxed state predispose to HCM, whereas mutations that affect the motor domain lead to DCM (27). MYH7 E848G affects neither: it is not predicted to affect the interacting heads motif or the motor domain. However, our data nevertheless strongly suggest that this is a pathogenic variant that leads to adult-onset, nondilated, nonhypertrophied systolic cardiomyopathy, potentially due to disruption of the MYH7 S2 and cMyBP-C C1C2 protein–protein interaction.

## Conclusions

Overall, patient-specific iPSC-CMs from a family with MYH7 E848G–induced systolic dysfunction along with isogenic control subjects recapitulate the contractile dysfunction that is characteristic of this cardiomyopathy. We predict that other mutations which result in a disruption of the S2:C1C2 interaction will also exhibit a similar phenotype, and that restoration of the protein–protein interaction would prove therapeutic.

Perspectives **COMPETENCY IN MEDICAL KNOWLEDGE:** Genetic cardiomyopathies resulting in systolic dysfunction can be due to MYH7 variants that impair contractile function on both cellular and tissue levels.

**TRANSLATIONAL OUTLOOK:** Further studies using our novel isogenic pluripotent stem cell-derived cardiomyocyte (MYH7 knockout) line will accelerate the screening of other MYH7 variants for potential disease relevance.
