# Supplementary material for: Novel Adult-Onset Systolic Cardiomyopathy Due to MYH7 E848G Mutation in Patient-Derived Induced Pluripotent Stem Cells
Source: JACC Basic Transl Sci. 2018 Dec 31;3(6):728–40. doi: 10.1016/j.jacbts.2018.08.008 (PMC6314962; doi:10.1016/j.jacbts.2018.08.008)
Supplement: Supplemental Data [file mmc1.pdf]

SUPPLEMENTAL MATERIALS

SUPPLEMENTAL METHODS

Patient Recruitment and Genetic Testing

Protocols for this study were approved by Seattle Children's Hospital Human Research Institutional Review Board. Written consent was obtained from all study participants. Comprehensive cardiac echocardiography including 2D-guided quantitative M-mode and spectral and tissue Doppler evaluations were performed on individuals Ic, WT IIa, FCM Ia, FCM IIb, and FCM IIc. Peripheral blood was drawn from FCM Ia and sent to Partners Healthcare Laboratory for Molecular Medicine (Cambridge, MA) in 2007 for direct exon sequencing of 9 genes (MYH7, MYBPC3, TNNT2, TNNI3, ACTC, MYL2, MYL3, LAMP2, and PRKAG2). When compared to a large number of controls, MYH7 E848G was reported as a private mutation by direct exon sequencing. Subsequent allelic analyses of the other family members identified concurrence between this heterozygous mutation and the presence of cardiomyopathy. The two unrelated study participants underwent allelic analysis and were found to not have the mutation.

Derivation of Patient-Specific iPSCs

Undifferentiated human IMR90 iPSCs, originally derived from fetal fibroblasts [1] (James A. Thomson, University of Wisconsin-Madison), were used as WT 3 iPSCs in this study. All other hiPSCs were established as follows:

A 3 to 5 mm skin punch biopsy was obtained from the forearm of all study participants. The human dermal fibroblasts (HDFs) were expanded and passaged twice prior to being

reprogrammed with episomal vectors expressing the transcription factors *OCT3/4, SOX2, KLF4, L-Myc, Lin28,* and *p53-shRNA*, according to published methods [2]. Episomal vectors, bearing human *OCT3/4, SOX2, KLF4, L-Myc, Lin28,* and *p53-shRNA* were obtained from Addgene (Plasmid 27077, 27078, and 27080). The expression plasmid mixture was electroporated with the Neon Transfection System (Invitrogen). Conditions used were 1,650 V, 10 ms, and 3 time pulses. Colonies were manually passaged onto a feeder layer of irradiated mouse embryonic fibroblasts (MEFs) and grown in MEF-conditioned medium supplemented with 10 ng/mL bFGF (Stemgent). Individual hiPSC clones with similar morphology to human ESCs were selected and further expanded on feeder cells. Only karyotypically normal hiPSCs were expanded and used for this study. All six patient-specific iPSC lines were characterized with pluripotency markers, SSEA4 (DSHB) and GCTM2 (gift from Dr. Martin Pera) by flow cytometry and by staining for alkaline phosphatase (Vector).

Generation of Patient-Specific iPSC-CMs

Human iPSCs were differentiated to cardiomyocytes using a monolayer platform as described previously[3,4] by induction with activin A (R&D Systems) and bone morphogenetic protein-4 (BMP4, R&D Systems) supplemented with the sequential activation and repression of the Wnt/β-catenin pathway using small molecules. Briefly, under serum-free monolayer culture conditions, hiPSC cultures were supplemented with Wnt agonist, 1 μM CHIR-99021 (Cayman chemical), for one day to reach confluence. The next day (day 0), the cells were induced to differentiate by replacing the media with RPMI (Invitrogen) supplemented with insulin-free B27 supplement (Life Technologies), 100 ng/ml activin A, and 1:60 diluted Matrigel (Fisher). The next day (day 1), the RPMI media was changed and supplemented with 5 ng/ml BMP4, 1 μM

CHIR-99021, and insulin-free B27. On day 3, the media was replaced with Wnt antagonist, 1 μM Xav-939 (Tocris). Cultures were fed on day 5 with RPMI plus insulin-free B27, and spontaneous beating was generally noted 7-10 days after activin A induction. Starting on day 7, the cultures were fed with RPMI supplemented with B27 containing insulin (Invitrogen). Only cell preparations >60% positively stained for cardiac troponin T (Thermo Scientific) were used for this study. Cells were cryopreserved as previously described [5], on day 20 of differentiation and subsequently thawed for single cell analysis. Cells used to generate the EHTs were replated at day 14 of differentiation and were never cryopreserved.

Fabrication of a Poly(urethane acrylate) Nanopatterned Substratum

A UV-curable poly(urethane acrylate) (PUA, Minutatek, Korea) master mold was fabricated using capillary force lithography as published previously [6-8] and was used as the nanopatterned template for all PUA substrata. Briefly, a glass coverslip (Ø18mm, Fisher) was cleaned using isopropyl alcohol and brush coated with an adhesion promoter (Glass Primer, Minuta Tech) and allowed to dry. 20 µl of PUA prepolymer (Norland Optical) was added to the coverslip and covered with the PUA master mold consisting of 800nm wide and 500nm deep parallel grooves and ridges, with the PUA prepolymer drawn into the nanofeatures via capillary force. The template-prepolymer-glass was cured via 365nm UV light to initiate photopolymerization for 5 min, the PUA master mold was then peeled off from the PUA substratum via forceps, and the substratum was UV cured overnight to finalize polymerization.

Optical Contraction Analysis

Cells were visualized using a Nikon TS100 inverted microscope coupled to an IonOptix videomicroscopy system (IonOptix) as previously described [9]. Briefly, day 20 cryopreserved cells were thawed onto fibronectin-coated (Invitrogen, 5 μg/cm2) nanopatterned surfaces at 10,000 cells/cm^2. Cells were cultured for 15 +/- 5 days (total 30-40 days with average age of 33 days) for the "Day 30" cells and 38 +/- 10 days (total 48-67 days with average age of 54 days) for the "Day 50" cells. All cells were paced at 1.5 Hz and perfused with 37° C HEPES-buffered Tyrodes solution ([10], 1.8 mM Ca^{2+}). Although not blinded, isolated contracting cells entrained at 1.5 Hz were randomly selected for analysis. Traces were collected and analyzed using the IonWizard software, and a minimum of five traces were analyzed and averaged for each cell.

Calcium Imaging

As previously described in our group, intracellular calcium content was measured with fura2-AM, a ratiometric indicator dye, on the IonOptix platform [9]. Briefly, day 20 cryopreserved cells were thawed onto fibronectin-coated (5 ug/cm2) glass coverslips at 60,000 cells/cm^2. Cells were cultured for 35 +/- 6 days (total 49-60 days with average age of 52 days) for the "Day 50" cells. On day of measurement, cells were incubated with 0.1 µM fura2-AM dye (Life Technologies) for 15 mins at 37° C and then washed with PBS. All cells were paced at 1.5 Hz and perfused with 37° C Tyrodes solution (1.8 mM Ca^{2+}). Only isolated contracting cells entrained at 1.5 Hz were selected for analysis. Calcium transients were measured with IonOptix Stepper Switch system coupled to a Nikon inverted fluorescence microscope. The fluorescence signal was acquired using a 40X Olympus objective, passed through a 510-nm filter, and then quantified using a photomultiplier tube.

Preparation of PDMS Molds and Generation of Engineered Heart Tissue (EHT) Constructs

Adobe Illustrator CS6 was used develop the 2D design of the negative template. The design was laser etched into a 6 mm sheet of cast acrylic. To make the PDMS molds, the Sylgard 184 Silicone Elastomer Kit (Dow Corning) was used with a 10:1 ratio of base to curing agent and placed in a vacuum chamber for 20 minutes to remove air bubbles. The elastomer was then poured into the acrylic templates and placed back into the vacuum chamber for 40 minutes. Remaining air bubbles were manually removed using a 30 gauge needle and the PDMS placed in a 65°C oven to cure overnight. The PDMS mold was carefully removed from the template and autoclaved before use with cells. Constructs were generated as previously described [11] using rat collagen type 1 (final concentration 1.5 mg/ml, Gibco), 11% mouse basement membrane extract (Geltrex, Invtrogen), and RPMI medium. Each 30 *u*l suspension contained 1 million day 14 cardiomyocytes (60-80% cTNT positive by flow cytometry) and 0.2 million HS-27a (human marrow stromal cells, [12]). Cell suspension was pipetted into one of the 4 troughs of the PDMS mold. After 1 hr incubation at 37° C, constructs were placed in RPMI medium supplemented with B27 and conditioned with static stress for 17-21 days prior to force measurements. Pins were placed above of the constructs to prevent them from floating up out of the molds. Cells used for EHT generation were never cryopreserved.

Tissue Construct Force Measurement

After removing the pins from the PDMS molds, constructs were attached to a force transducer (Aurora Scientific, model 400A) and a length controller (Aurora Scientific, model 308B) as previously described [11]. While paced at 1.5 Hz and perfused with 37° C Tyrodes with 0.4 mM

Ca^{2+}, force measurements of contracting constructs were continuously monitored as preparation length was changed by adjusting the position of the length-controller arm. The starting length was set at 5 mm, the initial length of the construct when cast in the PDMS mold. Preparation length was increased by 4% in a step-wise fashion, up to 140% length or until the construct broke. Passive tension and amplitude of the paced isometric twitch force were measured on 4 or more transients at 15 sec after each length change in a custom LabView program [11]. Active and passive forces were plotted with respect to length changes, and statistical differences between the linear regression lines were calculated with ANCOVA.

Immunohistochemistry and Immunocytochemistry

Cells were fixed with 4% paraformaldehyde for 10 minutes, permeabilized with 0.2% Triton X-100, blocked in 1.5% normal goat serum for 1 hr at room temperature, and then incubated overnight at 4° C with the following primary antibodies: α-actinin (Abcam 9465, 1:1000), FLAG (Sigma F7425, 1:1000), or mouse monoclonal anti-β-myosin heavy chain clone A4.951 (ATCC,1:1 dilution of hybridoma supernatant). Immunofluorescent secondaries included Alexa 488-, 594-, or 633-conjugated goat anti-mouse or goat anti-rabbit antibodies (Invitrogen, 1:100). Samples with F-actin staining were incubated with FITC-labeled phalloidin (Sigma, 1:100) for 1 hour at room temperature followed by a 5 minute Hoechst (Sigma, 1:1000) stain. EHTs were fixed in 4% paraformaldehyde for 10 minutes and then blocked in 5% normal goat serum with 0.2% Triton X-100 for 2 hours at room temperature. Alpha-actinin antibody was then added (1:20) overnight at 4° C. Samples were PBS washed for 24 hours and then probed with Alexa Fluor 594 goat anti-mouse secondary (1:200) and FITC-labeled phalloidin (1:100) for 2 hours at

room temperature. EHTs were again PBS washed for 24 hours prior to staining with Hoechst (1:1000) for 5 minutes and whole mounted onto a microscope slide.

Brightfield images were acquired with a Canon camera mounted onto Nikon Eclipse TS100 inverted microscope while confocal images were acquired with a Nikon A1R confocal microscope. Images were exported into Photoshop CS3 (Adobe) for processing. If necessary, brightness and contrast were adjusted for the entire image, and the image was cropped.

Quantitative analysis of cell alignment.

To assess alignment, immunofluorescent images of phalloidin-stained constructs were taken with the edges of the constructs present in each image, and analyzed using a modified, previously published MATLAB script utilizing pixel gradient analysis [13]. Briefly, the images are filtered, a gradient magnitude of each pixel in the image is calculated, and then the images are thresholded to determine the borders of the areas of interest. The orientation of the gradient was then calculated in respect to the edge of the construct, with the construct edge being the 0 degree angle. The gradient orientation angle for each pixel in a given, user-designated grid was then averaged with respect to the edge and plotted as a histogram.

Generation of E848G mutation

Full length human MYH7 cDNA (Thermo Scientific, BC112173) was subcloned into pShuttle-CMV (Ad-Easy). E848G mutation was created using a nested PCR technique with Phusion High-Fidelity DNA polymerase (NEB) according to the manufacturer's protocol. Amplicon 1 was created with primer 1 forward (ATCCGCATCTGCAGGAAAG) and primer 2 reverse (GGAGGCCATCCCCTTCTCT). Amplicon 2 was created with primer 2 forward

(CAGAAAGAGAGAAGGGGATGG) and primer 2 reverse (CTTCTCCTTGGTCAGCTTGG). Amplicon 1 and amplicon 2 were allowed to anneal and then amplified with primer 1 forward and primer 2 reverse. The final amplicon was subcloned back into pShuttle-CMV containing MYH7. The entire insert in both pShuttle-CMV MYH7 and pShuttle-CMV MYH7 E848G were confirmed with sequencing.

Generation of adenoviruses

GFP, c-term flag-tagged MYH7, and c-term flag-tagged MYH7 E848G were subcloned into the AdEasy pShuttle-CMV vector to generate Ad-GFP, Ad-MYH7, and Ad-E848G as described[14].

CRISPR/Cas9 Plasmid Construction and Transfection

To generate MYH7 knockout iPSCs (MYH7 KO iPSCs), we used CRISPR/Cas9 in WTC11 iPSCs (gift from Dr. Bruce Conklin, Gladstone Institute of Cardiovascular Disease[15]) to induce a double strand break shortly after the start codon in exon 3 of MYH7. Clones were screened to identify indel mutations that result in the creation of a new premature stop codon. Guide RNAs targeting the third exon of MYH7 were designed using the web tool http://crispr.mit.edu. Oligos for the guide targeting the sequence 5'-GATTCGGAGATGGCAGTCTT-3' were annealed and ligated into the BbsI digested pSpCas9(BB)-2A-Puro V2.0 vector (PX459) as described[16]. The vector was a gift from Feng Zhang (Addgene plasmid # 62988). Integration into the plasmid was confirmed by sequencing.

The WTC11 iPSC line was maintained on Matrigel in mTeSR™1 basal medium with supplement (Stemcell Technologies), which was exchanged every day. When the cells reached 60–70% confluency in 10 cm plates, the colonies were dissociated into single cells using

Versene EDTA-based cell dissociation reagent. 5 µg guide RNA-encoding plasmids were transfected into 1x106 iPS cells by electroporation using the the Amaxa™ Human Stem Cell Nucleofector™ Kit 1. The transfected cells were plated on Matrigel in 10 cm plates with mTeSR™1 basal medium with supplement (Stemcell Technologies). We added 10 µM Y-27632, a Rho-associated kinase (ROCK) inhibitor (Millipore), for 24 hrs after transfection to promote cell survival. 48 hrs post-transfection puromycin (0.5 ug/mL) was used to select for transfected cells for another 48 hrs. Between 7 and 10 days after puromycin selection, single colonies were picked and transferred into single wells of 24 well plates. After propagation, individual colonies were harvested. Three quarters of the cells were cryopreserved. One quarter was used to isolate genomic DNA using the DNeasy Blood & Tissue Kit (Qiagen) for sequencing.

Analysis of CRISPR/Cas9-mediated Indel Formation in MYH7 Gene and Screening for Off-Target Site Mutations

To analyze the indel formation in the MYH7 gene, the targeted region in exon 3 was PCR amplified using the primers: Forward 5'- GATTTCTGCTCCTACAGGGA-3' and Reverse 5'-GTTAGGAGTTGGTGAGTGACAG-3'. The PCR products were purified using the QIAquick PCR purification kit (Qiagen) and sequenced using the primer 5'-CTCCTCTGGTTAAGGGGTAA-3'. Cell lines with a confirmed new premature stop-codon in both alleles (homozygote) were identified (MYH7 KO iPSC), and clone 3b was used for subsequent experiments. All cell lines had normal karyotype after gene editing. The five possible off-target regions predicted by the website tool were also sequenced by PCR amplification of genomic DNA from each clonal line. Primer sequences for off-target region amplification are listed in table S3.

Western Blot

Protein was harvested using cold RIPA buffer containing protease and phosphatase inhibitors (Cell Signaling 9806). Lysate was clarified by centrifugation at 16,000g for 15 min at 4 °C, and protein concentration was assessed using the Pierce BCA Protein Assay Kit (ThermoFisher, 23225) according to the manufacturer's instructions. Protein was then solubilized in SDS-loading buffer and boiled for 5 mins. 10 μg of each protein sample was loaded onto 4–20% Mini-PROTEAN TGX Precast Protein Gels (Bio-Rad 4561094) and separated with electrophoresis. Proteins were transferred onto Immobilion-P PVDF membranes (Millipore Sigma IPVH00010) using a wet transfer system (Bio-Rad Laboratories). Membranes were blocked in TBS supplemented with 0.1% Tween-20 (hereafter TBST) and 5% milk for 1 h at room temperature. Primary antibody incubation was performed overnight at 4 °C under agitation in TBST supplemented with 5% bovine serum albumin. The following primary antibodies were used: 1:1,000 dilution of MYH7 (clone A4.95, Developmental Studies Hybridoma Bank); 1:1 dilution of MYH6 (clone BA-G5, Abcam ab50967); 1:10,000 dilution of GAPDH (Abcam ab8245), 1:300 dilution of alpha sarcomeric actinin (Abcam ab9465); 1:1,000 dilution of GFP (Novus Biologicals NB600); 1:1,000 dilution of FLAG (Sigma F7425). Secondary antibody dilution: 1:10,000.

Yeast Two-Hybrid Analysis

Yeast two-hybrid analysis was performed to study the effect of the E848G mutation in the distal region of human myosin S2 on its ability to interact with the C1-C2 region human cMyBP-C. The strategy employed is similar to that previously described [17]. In short, the region encoding the 126 distal amino acids of S2 of human beta myosin heavy chain, harboring the E848G mutation,

was PCR amplified using PfuUltra (Agilent Technologies) according to the manufacturer's protocol from amplified from pCMV-SchuttleMYH7E848G using the following primers: MYH7 GAD S2FIn-Fusion – GGAGGCCATGGAATTCATGCCGCTGCTGAAGAGTGCAG and MYH7 GAD S2RIn-Fusion – CGAGCTCGATGGATCCCTATTTGGCCAGTGTCAGCTCCAG. This PCR product was cloned into pGADT7 using the In-Fusion® HD cloning kit (Clontech) according to the manufacturer's protocol, giving rise to the pGADT7-S2E848G plasmid. This plasmid was sequenced prior to transformation into the Y187 yeast strain (Clontech) according to the manufacturer's LiAc protocol. Additionally, pGADT7, pGADT7-T, pGADT7-S2WT in Y187 yeast and pGBKT7, pGBKT7-53, pGBKT7-C1C2WT and pGBKT7C1C1E258K in Y2HGold yeast, described in de Lange et al. (2013) [17], was used in small scale yeast mating according to the manufacturer's protocol (Clontech). Following yeast mating between bait-containing Y2HGold and prey-containing Y187 yeast, diploid yeast colonies were selected on dropout selection media lacking leucine and tryptophan plates. We subsequently assayed for activation of the HIS3 and ADE2 nutritional reporter genes by sequentially plating diploid yeast colonies on dropout selection media lacking leucine, tryptophan and histidine (TDO) and on dropout selection media lacking leucine, tryptophan, histidine and adenine (QDO) plates. Growth on selection media was assessed and recorded 7 days post plating on respective media.

Statistics

Statistical differences between two groups were analyzed with unpaired two-tailed Student's t tests unless otherwise noted. Because the sample sizes were significantly larger in the single cell functional assays (Figure 2 and Figure 5), the Shapiro-Wilk normality test was applied and non-

parametric statistical tests (Kruskal-Wallis followed by a two-tailed Mann-Whitney U test) were subsequently performed if a Normal distribution was rejected.

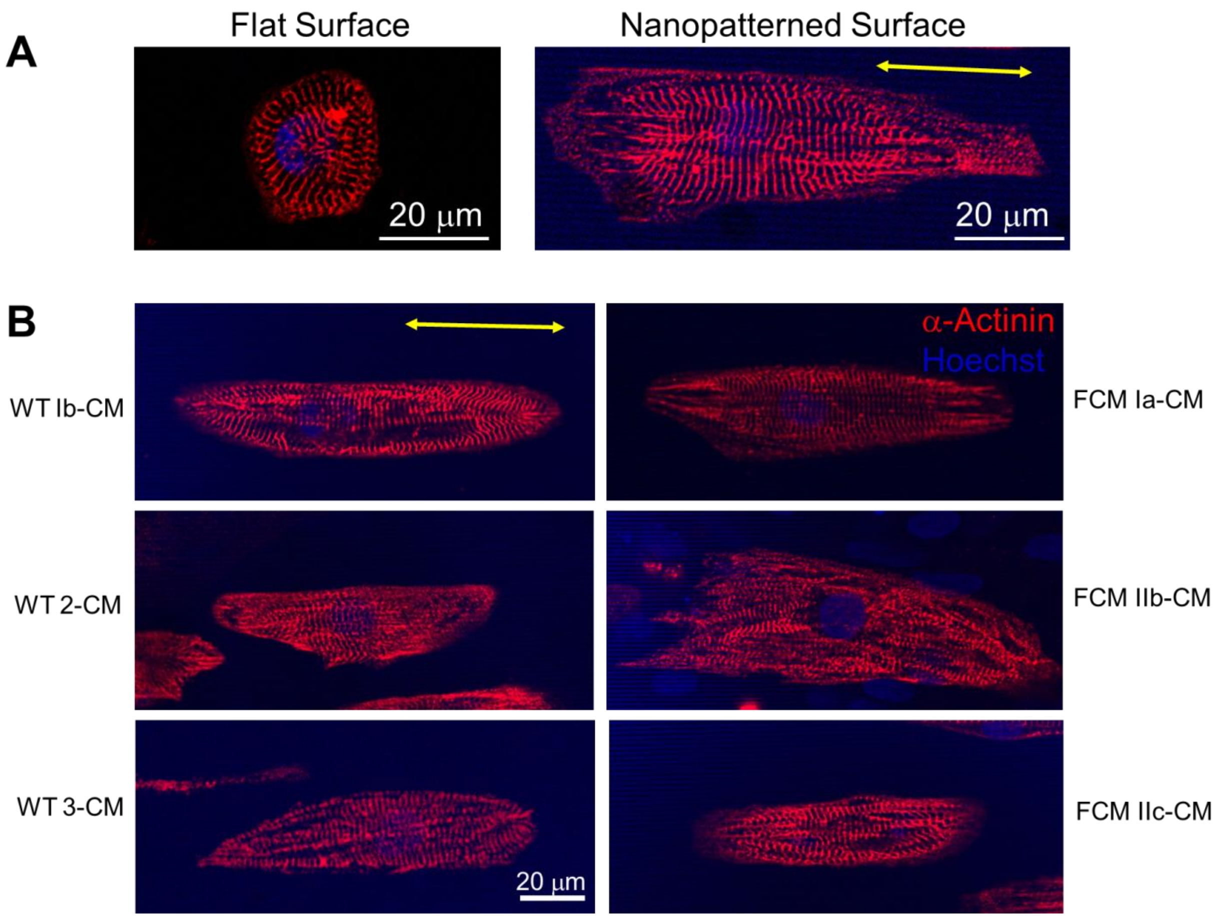

Supplemental Figure 1. Generation of patient-specific iPSC-CMs with aligned myofibrils. (**A**) HiPSC-CMs seeded on a nanopatterned surface for 5 days form significantly more aligned myofibrils when compared to age-matched cells replated on a flat surface. Double-headed yellow arrow in (A) and (B) denote the direction of anisotropic nanopatterns consisting of ridges and grooves. (**B**) Representative images of all six patient-specific iPSC-CMs seeded on nanopatterned surfaces for 30 days. Red, α-actinin; blue, Hoechst.

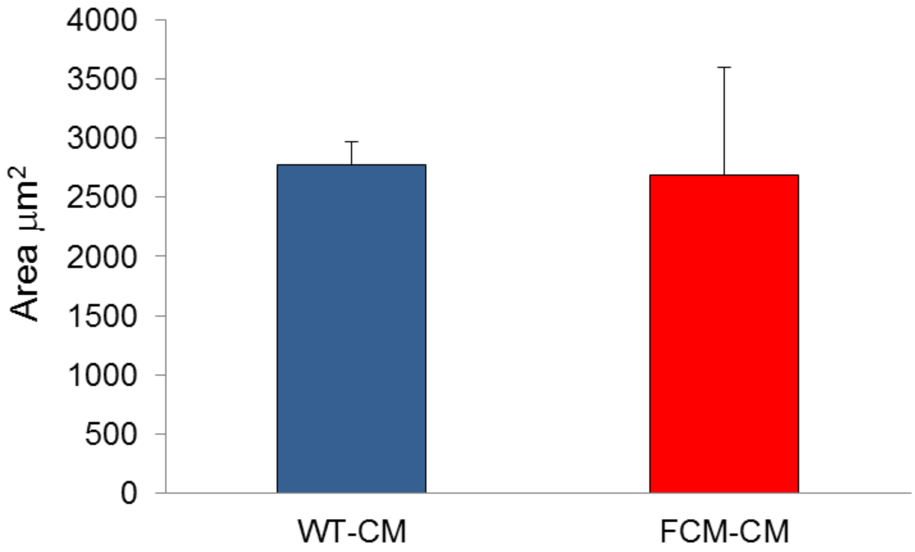

Supplemental Figure 2. Cell size quantification for three WT lines (WT Ib, WT 2, WT3) and three FCM lines (FCM Ia, FCM IIb, FCM IIc). 50 cells were analyzed for each line. Error bars = SEM.

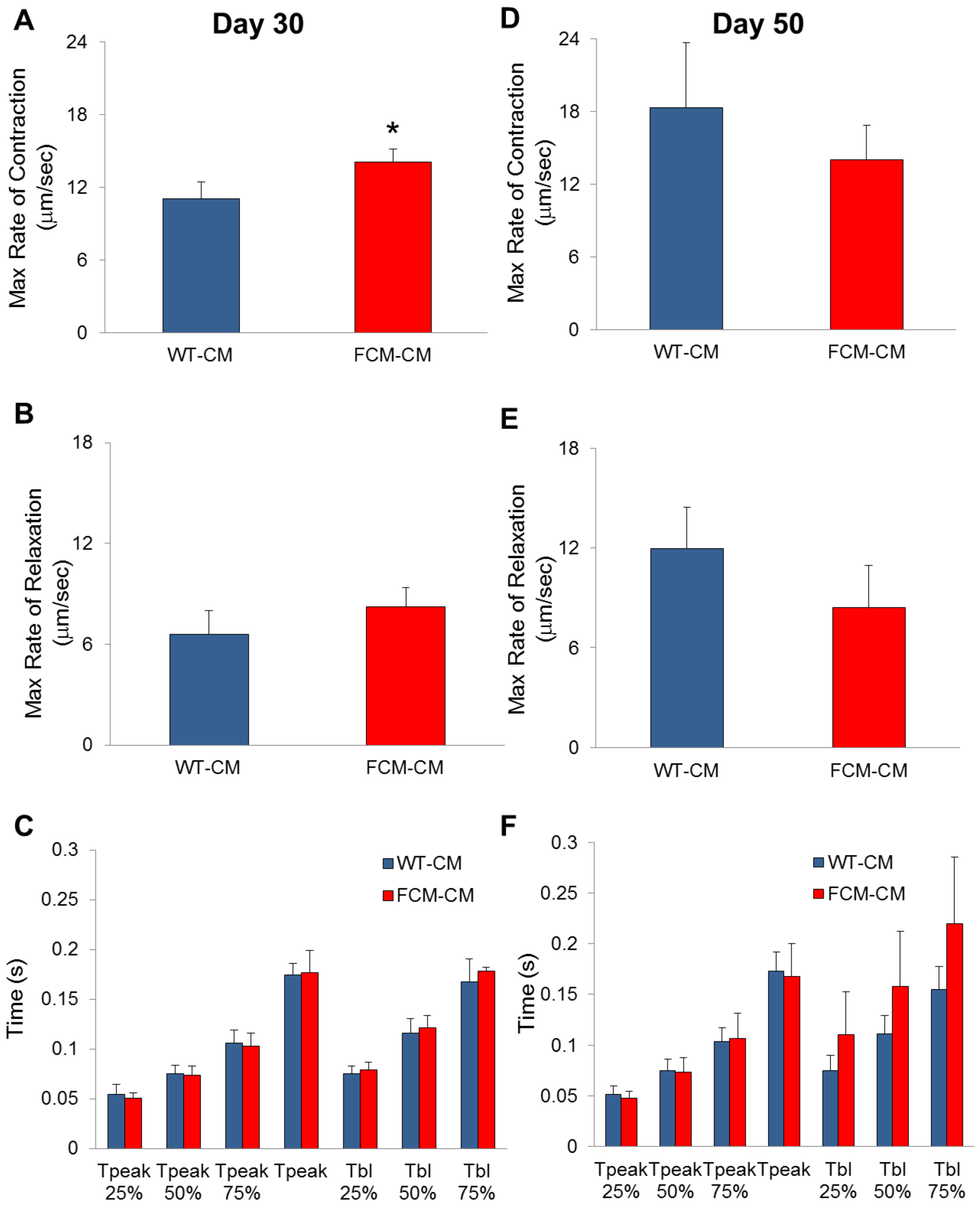
A
Day 30
24
18
12
6
0
Max Rate of Contraction (μm/sec)
*
WT-CM
FCM-CM
D
Day 50
24
18
12
6
0
Max Rate of Contraction (μm/sec)
WT-CM
FCM-CM
B
18
12
6
0
Max Rate of Relaxation (μm/sec)
WT-CM
FCM-CM
E
18
12
6
0
Max Rate of Relaxation (μm/sec)
WT-CM
FCM-CM
C
0.3
0.25
0.2
0.15
0.1
0.05
0
Time (s)
WT-CM
FCM-CM
Tpeak 25%
Tpeak 50%
Tpeak 75%
Tpeak
Tbl 25%
Tbl 50%
Tbl 75%
F
0.3
0.25
0.2
0.15
0.1
0.05
0
Time (s)
WT-CM
FCM-CM
Tpeak 25%
Tpeak 50%
Tpeak 75%
Tpeak
Tbl 25%
Tbl 50%
Tbl 75%

Supplemental Figure 3. Contractile characteristics of patient-specific iPSC-CMs. (A) Maximal rate of contraction was increased in FCM-CMs at day 30. (B) Max rate of relaxation, (C) time to [25, 50, 75%] peak and time to [25, 50, 75%] relaxation were not different. (D) Maximal rate of contraction, (E) maximal rate of relaxation, and (F) time to [25, 50, 75%] peak, and time to [25, 50, 75%] baseline were not different in the day 50 FCM-CMs. T_{peak} = time to peak contraction. Tbl = time to baseline (complete relaxation). Error bars (A,B,D,E) = St. Dev. Error bars (C, F) = SEM. *P<0.05 (Student's *t* test). WT-CM = average of means for WT Ib-CM, WT 2-CM, and WT 3-CM. FCM-CM = average of means for FCM Ia-CM, FCM IIb-CM, and FCM IIc-CM.

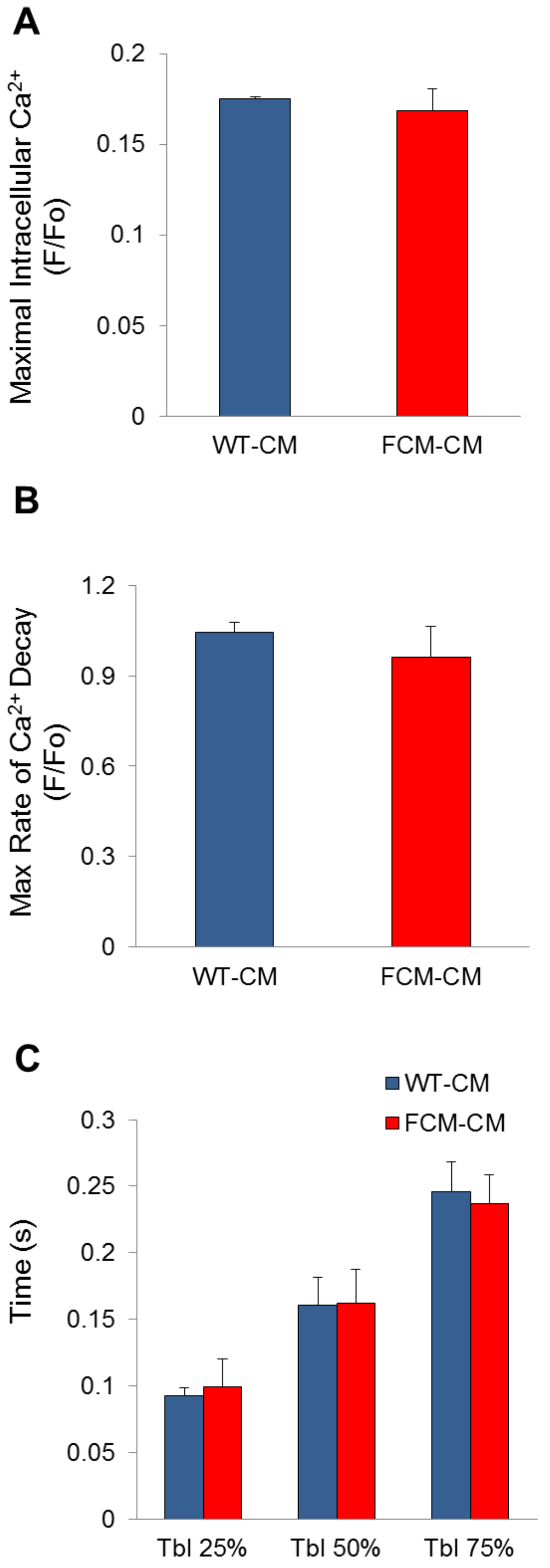
A
Maximal Intracellular Ca2+ (F/Fo)
0.2
0.15
0.1
0.05
0
WT-CM
FCM-CM
B
Max Rate of Ca2+ Decay (F/Fo)
1.2
0.9
0.6
0.3
0
WT-CM
FCM-CM
C
WT-CM
FCM-CM
Time (s)
0.3
0.25
0.2
0.15
0.1
0.05
0
Tbl 25%
Tbl 50%
Tbl 75%

Supplemental Figure 4. Calcium transient kinetics in patient-specific iPSC-CMs. Day 50 FCM-CMs did not exhibit any difference in max intracellular calcium (**A**), max rate of calcium decay (**B**), or time to [25, 50, 75%] calcium decay (**C**). WT-CM = average of means for WT 2-CM and WT 3-CM. FCM-CM = average of means for FCM Ia-CM and FCM IIc-CM. Error bars = SEM.

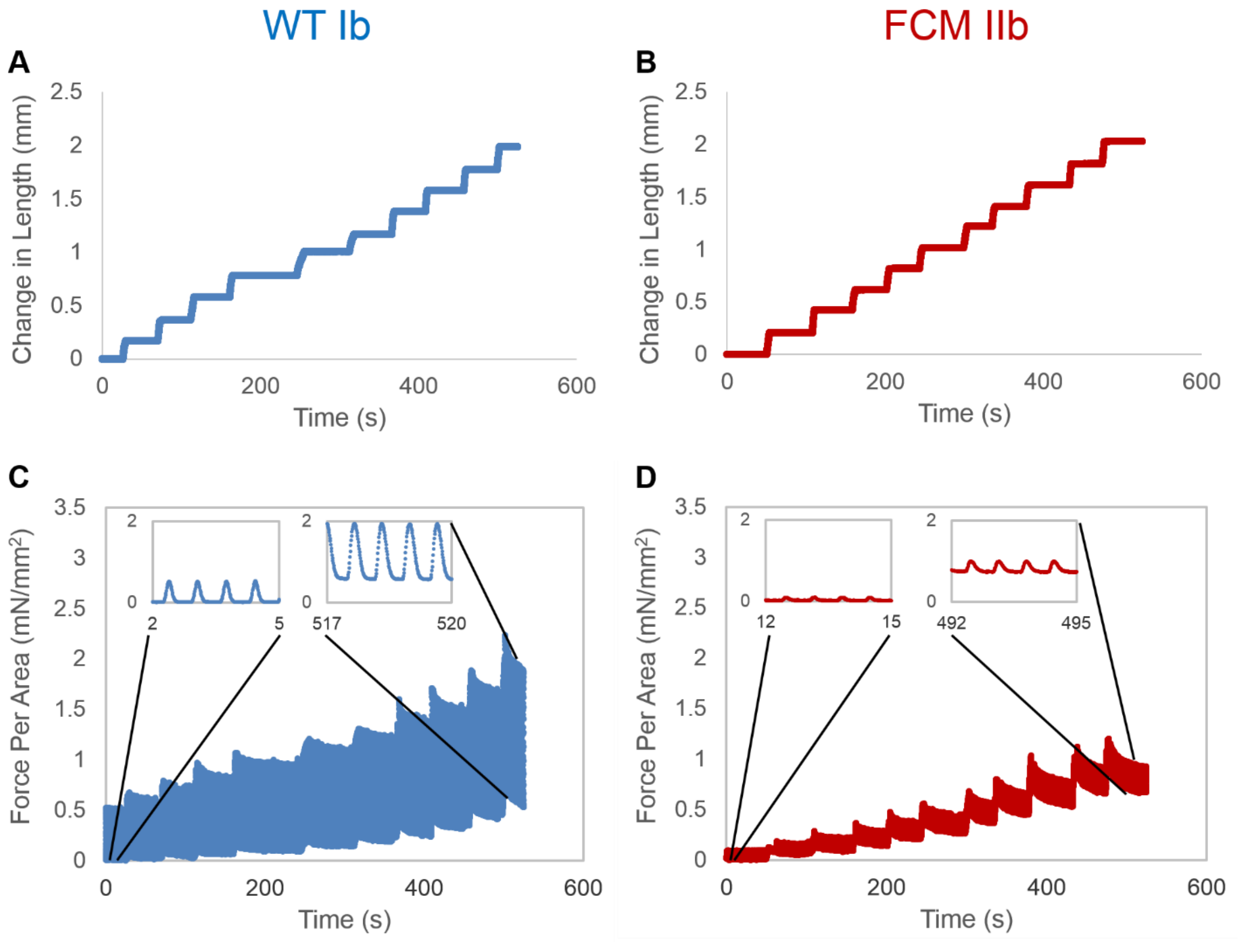

Supplemental Figure 5. Engineered heart tissues (5 mm preparation length) generated from patient-specific iPSC-CMs were conditioned for 2.5-3 weeks in static stress prior to assessment of active force development at various degrees of stretch. Representative WT Ib EHT and FCM IIb EHT tension traces are demonstrated here. EHTs underwent 10 serial steps of 4% length increase each to a maximum of 140% stretch (**A, B**). Force was continuously measured (**C, D**), and an increase in active force per area occurred when both WT Ib and FCM IIb EHTs were stretched (**insets**). For the same degree of stretch, the WT Ib EHT generated greater force than the FCM IIb EHT.

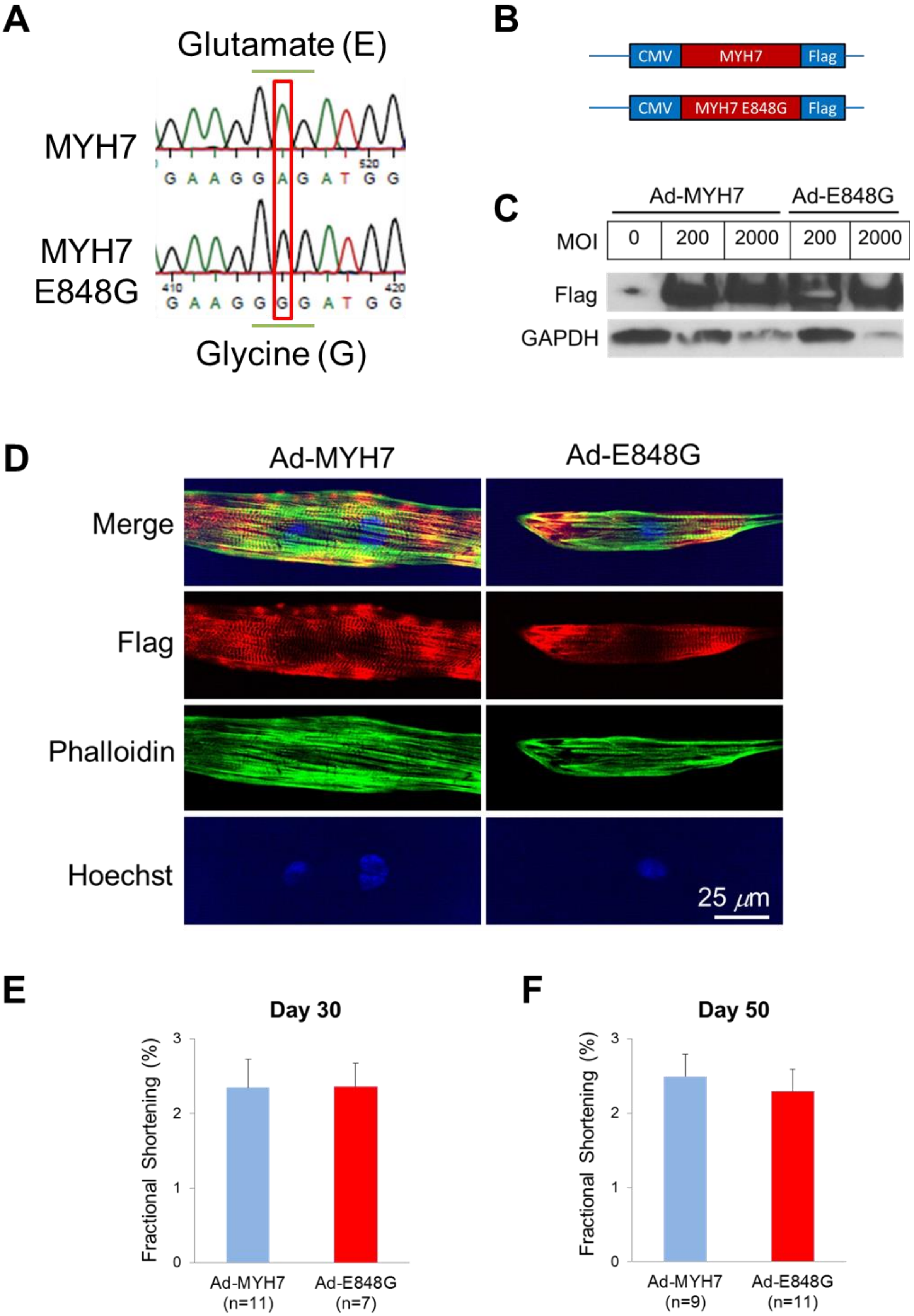
A
Glutamate (E)
MYH7
GAAGGAGATGG
MYH7 E848G
GAAGGGGATGG
Glycine (G)
B
CMV
MYH7
Flag
CMV
MYH7 E848G
Flag
C
Ad-MYH7
Ad-E848G
MOI
0
200
2000
200
2000
Flag
GAPDH
D
Ad-MYH7
Ad-E848G
Merge
Flag
Phalloidin
Hoechst
25 μm
E
Day 30
Fractional Shortening (%)
Ad-MYH7 (n=11)
Ad-E848G (n=7)
F
Day 50
Fractional Shortening (%)
Ad-MYH7 (n=9)
Ad-E848G (n=11)

Supplemental Figure 6. Generation of MYH7 and MYH7 E848G adenoviruses for functional studies (Ad-MYH7, Ad-E848G). (A) DNA sequencing confirms successful generation of MYH7 E848G mutation. (B) C-term flag-tagged MYH7 and MYH7 E848G are subcloned into the AdEasy pShuttle-CMV vector to generate Ad-MYH7 and Ad-E848G. (C) Immunoblot confirms predicted full length MYH7 protein expression with increased cytotoxicity at higher viral MOI (entire well of cell lysate loaded in each lane). (D) Immunocytochemistry demonstrates sustained incorporation of flag-tagged MYH7 and MYH7 E848G into sarcomeres of WT Ib-CMs transduced with 100 MOI of Ad-MYH7 or Ad-E848G after a period of 3 weeks. Fractional shortening is not significantly changed in WT Ib-CMs transduced with Ad-E848G compared to Ad-MYH7 after 30 days (E) or 50 days (F). All cells were paced at 1.5 Hz. Error bars indicate SEM.

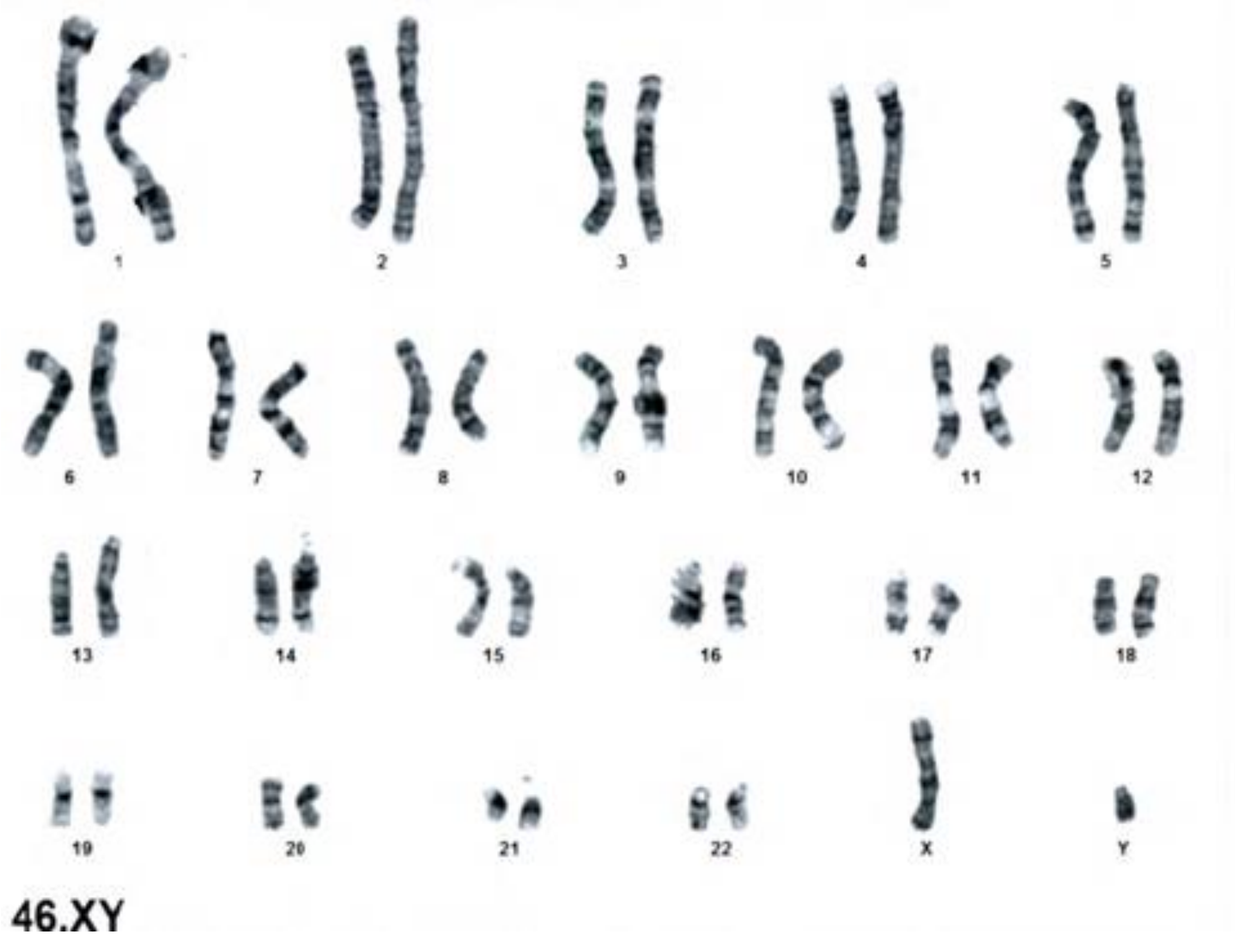

Supplemental Figure 7. Karotypically normal (46 XY) WTC MYH7 KO iPSC clone 3b.

Supplemental Table 1. Patient Characteristics

Patient	Ia	Ic	IIa	IIb	IIc	WT 2	WT3
MYH7 E848G mutation	+	?	-	+	+	-	-
Age	56	63	18	19	20	55	Fetal
Gender	M	F	M	M	F	M	F
NYHA class	III-IV	IV	-	I	I	-	-
Coronary artery disease	No	No	U	U	U	No	-
Hypertension	Yes	No	No	No	No	No	-
Hyperlipidemia	Yes	No	No	No	No	No	-
Diabetes	Yes	No	No	No	No	No	-
Tobacco	Never	Never	Never	Never	Never	Never	-

F = female; M = male, U = unknown, WT = wildtype

Supplemental Table 2. Echocardiographic Characteristics

**Patient**	**Ia**	**Ia**	**Ic**	**IIa**	**IIb**	**IIc**
**Age at time of echo**	**54**	**56**	**63**	**18**	**19**	**20**
**MYH7 E848G mutation**	**+**	**+**	**?**	**-**	**+**	**+**
LVEF (%) [nl 55-75%]	28	25-30	31	65	66	70
IVS wall thickness at end diastole (mm)	13	10	11	9	9.9	8.8
IVS wall thickness at end systole (mm)		12	14	16	13.6	10.4
IVS systolic thickening (%) [nl 53 +/- 13%] [18]		20.0	27.3	77.8	37.4	18.2
PW thickness at end diastole (mm)	10	11	12	10	9.6	6.8
PW thickness at end systole (mm)		15	21	15	13.2	12.1
PW thickening (%) [nl 58 +/- 13%] [19]		36.4	75.0	50.0	37.5	77.9
LVEDD (mm) [nl 38-56 mm]	44	37	42	55	55	38
LVEDD/BSA (cm/m2) [nl 2.2-3.1]	2.1	1.8	2.2		3.0	2.5
LV volume index (mL/m^2) [nl 35-75 mL/m^2]		34	22		58	59

IVS = interventricular septum; LVEDD = left ventricular end diastolic dimension; LVEF = left ventricular ejection fraction; LVESD = left ventricular end systolic dimension; nl = normal; PW = posterior wall

Supplemental Table 3. Multiple Protein Sequence Alignment Surrounding the MYH7 E848 Locus

Human	A	E	R	E	K	**E**	M	A	S	M	K
Chimpanzee	A	E	R	E	K	**E**	M	A	S	M	K
Dog	A	E	T	E	K	**E**	M	A	T	M	K
Mouse	A	E	T	E	K	**E**	M	A	T	M	K
Conserved	A	E	-	E	K	**E**	M	A	-	M	K

MYH7 E848 locus (red) is conserved across multiple species.

Supplemental Table 4. Sarcomeric Alignment on Nanopatterned Surface

	**Average Angle of Sarcomeres**
**WT Ib-CM**	4.1 +/- 0.5
**WT2-CM**	8.7 +/- 1.1
**WT3-CM**	7.0 +/- 1.0
**FCM Ia-CM**	5.7 +/- 0.8
**FCM IIb-CM**	14.0 +/- 0.9
**FCM IIc-CM**	6.7 +/- 0.9

For every hiPSC-CM line, the angle of deviation of the myofibrils from the anisotropically nanopatterned substratum was measured in at least 120 myofibrils from 30 different hiPSC-CMs. Error = SEM.

Supplemental Table 5. Primer Sequences for CRISPR/Cas 9 Off-Target Region Amplification

NM_175737<br>("KLB")	Fwd.: TGTAACAACCTGGAGAATCACA<br>Rev.: CCAAGCTGGTCTCAAACTCCT
NM_001204185<br>("TP73")	Fwd.: TGCAGAACCTTCTGGAGCTG<br>Rev.: GTTGTTGTTAGGGGCAGGAA
NM_024996<br>("GFM1")	Fwd.: ACTTTGACGCGTGCTCTG<br>Rev.: ACTGCACATCCAAAATGCAC
NM_152562<br>("CDCA2")	Fwd.: TTTGGATTCAATGGCTGTAGG<br>Rev.: CCATGCGGAAATGTAGGATT
NM_003959<br>("HIP1R")	Fwd.: TCCTTCATGGGAGCATCTG<br>Rev.: TGGGCAGTGGGTTTATTACTG

Supplemental Video 1

IonOptix recording of a hiPSC-CM plated on nanopatterned surface 1.

Supplemental Video 2

IonOptix recording of a hiPSC-CM plated on nanopatterned surface 2.

Supplemental Video 3

MYH7 KO-CMs spontaneously beating.

SUPPLEMENTAL REFERENCES

1. Yu J, Vodyanik MA, Smuga-Otto K, et al. Induced pluripotent stem cell lines derived from human somatic cells. Science. 2007;318(5858):1917-1920.
2. Okita K, Matsumura Y, Sato Y, et al. A more efficient method to generate integration-free human iPS cells. Nat Methods. 2011;8(5):409-412.
3. Palpant NJ, Pabon L, Roberts M, et al. Inhibition of beta-catenin signaling respecifies anterior-like endothelium into beating human cardiomyocytes. Development. 2015;142(18):3198-3209.
4. Palpant NJ, Pabon L, Friedman CE, et al. Generating high-purity cardiac and endothelial derivatives from patterned mesoderm using human pluripotent stem cells. Nat Protoc. 2017;12(1):15-31.
5. Chong JJ, Yang X, Don CW, et al. Human embryonic-stem-cell-derived cardiomyocytes regenerate non-human primate hearts. Nature. 2014;510(7504):273-277.
6. Kim D-H, Seo C-H, Han K, Kwon KW, Levchenko A, Suh K-Y. Guided Cell Migration on Microtextured Substrates with Variable Local Density and Anisotropy. Advanced Functional Materials. 2009;19(10):1579-1586.
7. Kim D-H, Han K, Gupta K, Kwon KW, Suh K-Y, Levchenko A. Mechanosensitivity of fibroblast cell shape and movement to anisotropic substratum topography gradients. Biomaterials. 2009;30(29):5433-5444.
8. Kim P, Kim DH, Kim B, et al. Fabrication of nanostructures of polyethylene glycol for applications to protein adsorption and cell adhesion. Nanotechnology. 2005;16(10):2420-2426.
9. Lundy SD, Zhu WZ, Regnier M, Laflamme MA. Structural and functional maturation of cardiomyocytes derived from human pluripotent stem cells. Stem Cells Dev. 2013;22(14):1991-2002.
10. Korte FS, Dai J, Buckley K, et al. Upregulation of cardiomyocyte ribonucleotide reductase increases intracellular 2 deoxy-ATP, contractility, and relaxation. J Mol Cell Cardiol. 2011;51(6):894-901.
11. Tulloch NL, Muskheli V, Razumova MV, et al. Growth of Engineered Human Myocardium With Mechanical Loading and Vascular Coculture / Novelty and Significance. Circulation Research. 2011;109(1):47-59.
12. Kreutziger KL, Muskheli V, Johnson P, Braun K, Wight TN, Murry CE. Developing vasculature and stroma in engineered human myocardium. Tissue Eng Part A. 2011;17(9-10):1219-1228.
13. Jiao A, Trosper NE, Yang HS, et al. Thermoresponsive nanofabricated substratum for the engineering of three-dimensional tissues with layer-by-layer architectural control. ACS Nano. 2014;8(5):4430-4439.
14. He T-C, Zhou S, da Costa LT, Yu J, Kinzler KW, Vogelstein B. A simplified system for generating recombinant adenoviruses. Proceedings of the National Academy of Sciences. 1998;95(5):2509-2514.
15. Miyaoka Y, Chan AH, Judge LM, et al. Isolation of single-base genome-edited human iPS cells without antibiotic selection. Nature methods. 2014;11(3):291-293.
16. Ran FA, Hsu PD, Wright J, Agarwala V, Scott DA, Zhang F. Genome engineering using the CRISPR-Cas9 system. Nature protocols. 2013;8(11):2281-2308.
17. De Lange WJ, Grimes AC, Hegge LF, Spring AM, Brost TM, Ralphe JC. E258K HCM-causing mutation in cardiac MyBP-C reduces contractile force and accelerates twitch kinetics by disrupting the cMyBP-C and myosin S2 interaction. J Gen Physiol. 2013;142(3):241-255.

18. St John Sutton MG, Tajik AJ, Mercier LA, Seward JB, Giuliani ER, Ritman EL. Assessment of left ventricular function in secundum atrial septal defect by computer analysis of the M-mode echocardiogram. Circulation. 1979;60(5):1082-1090.

19. Palka P, Lange A, Fleming AD, et al. Differences in myocardial velocity gradient measured throughout the cardiac cycle in patients with hypertrophic cardiomyopathy, athletes and patients with left ventricular hypertrophy due to hypertension. J Am Coll Cardiol. 1997;30(3):760-768.
